# FATP2-mediated lipid metabolism enhances chimeric antigen receptor T-cell therapy resistance in B-cell acute lymphoblastic leukemia

**DOI:** 10.1038/s41375-026-03030-0

**Published:** 2026-06-30

**Authors:** Clarissa Garcia, Kaylyn U. Lyons, Julian Grandvallet Contreras, Tian Liu, Alexis J. Donnelly, Amanda J. Novak, Amy Argabright, Colin Anderson, Abby Grier, Sabrina Smith, Joshua Michlin, Jesutomisin Olusoji, Railey G. Mikeska, Xin Zhou, Huimin Geng, Jeremy T. Rahkola, Hiten N. Patel, Jeffrey G. Jacot, Markus Müschen, Angelo D’Alessandro, John E. Dick, Ilaria Iacobucci, Charles G. Mullighan, Julie A. Reisz, Tzu Phang, M. Eric Kohler, Matthew T. Witkowski

**Affiliations:** 1 Department of Pediatrics, University of Colorado Anschutz Medical Campus, Aurora, CO USA; 2 Department of Biochemistry and Molecular Genetics, University of Colorado Anschutz Medical Campus, Aurora, CO USA; 3 Department of Molecular Genetics, University of Toronto, Princess Margaret Hospital, University Health Network, Princess Margaret Cancer Research Tower, Toronto, ON Canada; 4 Department of Pathology, St Jude Children’s Research Hospital, Memphis, TN USA; 5 Department of Laboratory Medicine, University of California San Francisco, San Francisco, CA USA; 6 Rocky Mountain Regional Veteran Affairs Medical Center, Aurora, CO USA; 7 Department of Bioengineering, University of Colorado Anschutz Medical Campus, Aurora, CO USA; 8 Division of Pediatrics, Children’s Hospital Colorado, Aurora, CO USA; 9 Center of Molecular and Cellular Oncology, Yale Cancer Center, Yale University, New Haven, CT USA; 10 Division of Blood and Marrow Transplant & Cellular Therapeutics, Center for Cancer and Blood Disorders, Children’s Hospital Colorado, Aurora, CO USA; 11 Division of Hematology, University of Colorado Anschutz Medical Campus, Aurora, CO USA

**Keywords:** Cancer metabolism, Acute lymphocytic leukaemia

## Abstract

Relapsed/refractory B-cell acute lymphoblastic leukemia (B-ALL) remains a leading cause of cancer-related death in children and young adults. While CD19-directed chimeric antigen receptor T cell (CAR-T) therapy offers promise, high rates of long-term failure underscore the need to understand resistance mechanisms. Our studies found p53 inactivation promotes CAR-T resistance in human pre-B-ALL cell lines. Through genome-wide CRISPR/Cas9 screening of CAR-sensitive *TP53*-wildtype and CAR-resistant *TP53*-mutated CD19 + B-ALL cell lines, we found the Fatty Acid Transport Protein 2 (FATP2, encoded by *SLC27A2*) is a leukemia-intrinsic mechanism of CAR-T resistance in *TP53*-mutant B-ALL. High *SLC27A2* expression in pediatric B-ALL patients correlate with worse survival outcomes following conventional chemotherapy. Using B-ALL cell lines and patient-derived xenografts, we show that FATP2-expressing *TP53*-mutant B-ALL resistance to CAR-T is dependent on exogenous lipid uptake to fuel fatty acid oxidation (FAO) and cell survival, which can be pharmacologically targeted through inhibition of neutral lipolysis and CPT1. These findings identify FATP2-mediated fatty acid uptake and downstream FAO as a potential target to improve existing CAR-T efficacy in human B-ALL.

## Introduction

CAR-T cell therapy is highly effective at inducing remissions in relapsed/refractory B-ALL. Critically, for those patients who initially respond to CAR-T therapy (~80–90%), ~50% will relapse within one year of T cell infusion [1–5]. While short duration of CAR-T cell persistence and loss of CD19 antigen on B-ALL blasts have been linked to CD19-directed CAR-T relapse, these mechanisms do not account for all CAR-T failures [1, 2, 6–12]. The current CAR-T paradigm suggests CAR-T cells overcome chemotherapy resistance by killing CD19+ relapsed/refractory B-ALL in a T cell cytotoxicity-dependent manner that does not rely on the genotoxic or metabolic insults elicited by chemotherapy; however, emerging clinical evidence suggests additional leukemia-intrinsic factors contributing to chemotherapy resistance may be linked to suboptimal CAR-T responses. *TP53* (encoding p53) mutations are associated with dismal B-ALL patient outcomes with chemotherapy [13–15]; and recent clinical studies suggest *TP53* mutations predict inferior outcomes following CD19 CAR-T treatment for B-ALL [16–18] and B cell lymphoma [19]. Therefore, there is an urgent need to understand how leukemia-intrinsic resistance mechanisms, such as *TP53* mutations, contribute to disease progression after CAR-T therapy. Consistent with recent findings [18], we show that *TP53* mutations in human B-ALL cell lines increase resistance to CD19 CAR-T cell killing. Using genome-wide CRISPR screening approaches, we identify Fatty Acid Transport Protein 2 (FATP2) as a critical mediator of CAR-T resistance in *TP53*-mutant B-ALL. Using human B-ALL cell lines and patient-derived xenografts, we demonstrate that *TP53*-mutated B-ALL exhibits a significant dependence on exogenous fatty acid uptake via and downstream fatty acid oxidation (FAO) to resist CD19 CAR-T killing, which can overcome by pharmacological FAO inhibition.

## Results

### *TP53* mutations promote CAR-T resistance in human B-ALL cell line 697, but not NALM-6

Although *TP53* mutations are rare at pediatric B-ALL diagnosis, with the exception of low hypodiploid B-ALL cases [20], relapsed and/or chemotherapy-refractory B-ALL often harbor *TP53* mutations [13], which have been associated with CD19 CAR-T resistance [16–18]. To test the effect of leukemia cell p53 activity on CAR T cell effectiveness, we generated isogenic human CD19^+^ B-ALL cell lines, 697 (*TCF3::*PBX1) and NALM-6 (*DUX4-*rearranged), harboring *TP53*-wildtype (*TP53*^+/+^), a single allele *TP53* “hotspot” mutation (the p.G245D DNA-binding domain mutant, herein referred to as *TP53*^G245D/+^), or *TP53* frameshift mutations (*TP53*^+/−^ or *TP53*^−/−^). *TP53* frameshift mutations were introduced into Cas9-expressing B-ALL via lentiviral transduction of sgRNA constructs targeting *TP53*, tracked by mCherry (mCherry/*TP53*−/− or mCherry/*TP53*+/−), with non-targeting sgRNA controls (sgROSA tracked by BFP, BFP/*TP53*+/+). Single-cell clones were expanded for further experiments (Supplementary Fig. 1A). To confirm genetic ablation disabled p53 function, we treated clonally derived B-ALL with Nutlin-3A - a selective MDM2 inhibitor that prevents p53 degradation leading to p53-dependent apoptosis [21] - for 4 h followed by immunoblot analysis of p53 and p21, a direct target of p53. Nutlin-3A treatment induced expression of p53 in sg*ROSA*-expressing 697-BFP/*TP53*^+/+^ and NALM-6-BFP/*TP53*^+/+^; while sg*TP53*-transduced counterparts failed to display high levels of either p53 or p21, indicative of disrupted p53 activity (Supplementary Fig. 1B). *TP53*-targeted amplicon sequencing (not shown) revealed a homozygous frameshift in NALM-6-mCherry/*TP53*^−/−^, whereas 697 sg*TP53*-mCherry harbored a heterozygous frameshift mutation (mCherry/*TP53*^+/−^) with limited p53 induction and no p21 induction in Nutlin-3a-treated cells (Supplementary Fig. 1B). In addition to *TP53* genetic ablation, we modeled a *TP53* DNA-binding mutations observed in high-risk B-ALL [22]. Using an electroporation-based, transient CRISPR homology-directed repair strategy, we successfully introduced a single *TP53* c.734Gly>Asp (chr17:7577547:C/T, Assembly GRCh37) non-synonymous mutation into Cas9-expressing NALM-6 and 697 cells (herein referred to as *TP53*^.G245D/+^), confirmed by amplicon sequencing (not shown). We tested if p53 loss conferred Nutlin-3a resistance in B-ALL using a competitive growth assay, by mixing *TP53*-wildtype and *TP53*-mutant *(TP53*^+/−^ or *TP53* ^G245D/+^) isogenic 697 B-ALL lines at a ~2:1 ratio, respectively, and Nutlin-3a treatment for 72 h, then analyzed cultures by flow cytometry for changes in the proportion of *TP53-*mutant cells versus vehicle (DMSO)-treated controls. We found a significant survival advantage of *TP53*-mutant 697 leukemic cells at the expense of *TP53*-wildtype counterparts (Supplementary Fig. 1C, D) indicative of impaired p53-dependent apoptosis for *TP53*-mutant B-ALL.

**Fig. 1:**
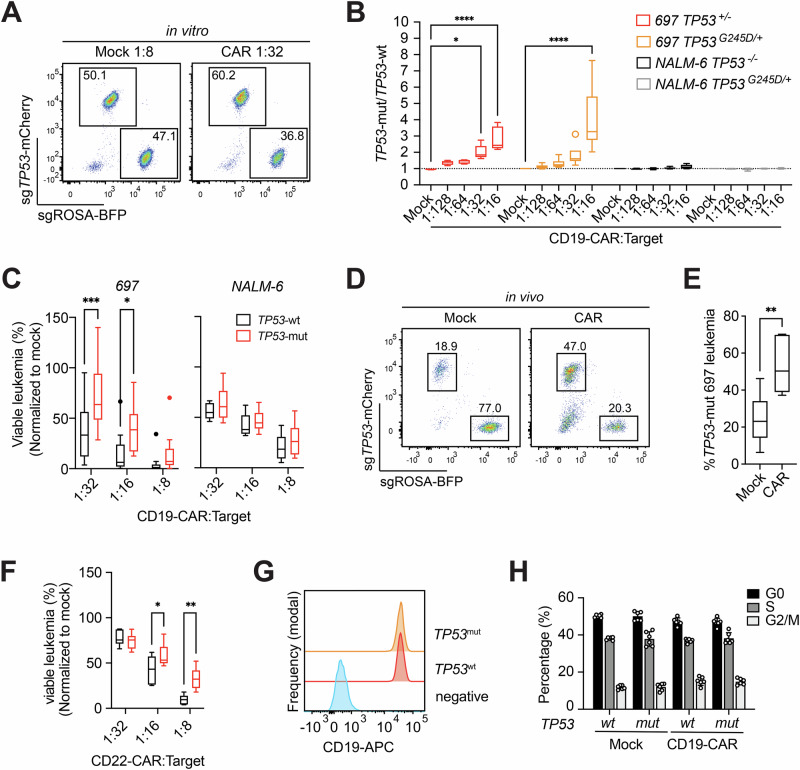
*TP53* mutations confer resistance to 697, but not NALM-6, B-ALL cell lines challenged with CD19-directed CAR-T cells. **A**, **B** Representative flow cytometry plots and histograms showing 697 *TP53*-mutant (+/–, G245D/+) to *TP53*-wildtype cell ratios after 72 h CAR-T or Mock T-cell treatment, normalized to Mock T-cell controls. Mock T cells were used at the highest E:T ratio (1:8) to account for maximal allogeneic reactivity. *TP53* genotype and CAR:target ratios are indicated. 2-way ANOVA with Dunnett’s multiple comparisons: *p < 0.05, **p < 0.01, ***p < 0.005, ****p < 0.001. *n* = 3–6 experiments; Tukey box-and-whisker plots. **C** Viable leukemia counts following repeated 72 h CD19-CAR-T challenges, normalized to Mock T-cell controls, for 697 (left) and NALM-6 (right) isogenic B-ALL lines with indicated *TP53* status. 2-way ANOVA, Dunnett’s multiple comparisons: *p < 0.05, **p < 0.01. *n* = 5 (NALM-6) and *n* = 9 (697); Tukey box-and-whisker plots. **D** Representative flow cytometry of *TP53*-mutant (mCherry+) and *TP53*-wildtype (BFP+) 697 cells in NSG bone marrow ~4 weeks after Mock or CAR-T infusion (5 × 10⁵ T cells, 4-day engraftment). **E** Percent mCherry+ *TP53*-mutant 697 cells in bone marrow. 2-way ANOVA: **p < 0.01. Six mice/group (3 male, 3 female); Tukey box-and-whisker plots. **F** Viable leukemia counts following repeated 72 h CD22-CAR-T challenges, normalized to Mock T-cell controls, for 697 isogenic B-ALL lines with indicated *TP53* status. 2-way ANOVA, Dunnett’s multiple comparisons: *p < 0.05, **p < 0.01. *n* = 6 replicates; Tukey box-and-whisker plots. **G** Surface CD19 expression histogram in *TP53*-mutant and *TP53*-wildtype 697 B-ALL cells. **H** Flow-based DNA content cell cycle phase quantification histogram in *TP53*-mutant and *TP53*-wildtype 697 cells co-cultured with Mock-T or CAR-T (1:16 E:T ratio).

We then tested if p53 loss or mutation conferred a survival advantage to B-ALL challenged with CAR-T cells. Using a competitive growth assay, we co-cultured a 1:1 mixture of isogenic *TP53*-mutant *(TP53*^+/−^, *TP53*^−/−^ or *TP53* ^G245D/+^) and *TP53*-wildtype cells from CD19-expressing 697 or NALM-6 lines with serial dilutions of CD19-directed CAR-T cells and mock T cell controls for 72 h. CAR-T cells generated by lentiviral transduction of peripheral blood T cells from healthy donors, using a construct encoding a CD19 CAR incorporating the FMC63 single-chain variable fragment (scFv) and a 4-1BB costimulatory domain. Successful transduction was confirmed by flow cytometry, using non-transduced (Mock) T cells as negative controls. We then analyzed the cultures by flow cytometry to quantify changes in the *TP53*-mutant:*TP53*-wildtype ratio. We then normalized CAR-treated ratios to mock T-cell-treated ratios to account for differences elicited by donor T-cell allo-reactivity. We found a dose-dependent positive selection of *TP53*^+/−^ or *TP53*^G245D/+^ 697 B-ALL over their *TP53*-wildtype counterparts after CAR T cell treatment (Fig. 1A, B, Supplementary Fig. 2A). Given the heterozygous *TP53* frameshift mutation in 697 cells, we examined whether homozygous *TP53* frameshift deletion affects 697 B-ALL resistance to CAR-T–mediated killing. Both *TP53*^+/–^ and *TP53*^–/–^ cells exhibited similar levels of CAR-T resistance compared to *TP53*^+/+^ controls (Supplementary Fig. 2B); however, *TP53*^–/–^ cells showed significantly reduced growth, as measured by cell counts and metabolic activity, relative to *TP53*-wildtype controls (Supplementary Fig. 2C, D). Therefore, all subsequent experiments were performed using *TP53*^+/–^ frameshift mutants (herein ‘*TP53*-mutant’). Notably, we saw no significant advantage of *TP53-*mutant NALM-6 versus *TP53*-wildtype (Fig. 1B). To confirm target-intrinsic CAR-T resistance in vitro, *TP53*-mutant or *TP53*-wildtype 697 and NALM-6 B-ALL were co-incubated with serial dilutions of CD19 CAR. Consistent with competition assays, we found reduced CD19 CAR sensitivity of *TP53*-mutant 697, but not *TP53*-mutant NALM-6, relative to *TP53*-wildtype counterparts (Fig. 1C).

We then modeled the impact of CAR-T pressure on *TP53*-mutant leukemia cells in vivo, in isogenic cell lines derived from CD19^+^ 697 B-ALL cells by mixing *TP53*-wildtype & *TP53*-mutant at 3:1, then transplanting into NOD-scid IL2Rg^null^ (NSG) recipient mice. At 4 days following engraftment, we split the cohort into two groups, and infused recipients with a subtherapeutic dose of CD19 CAR T cells or mock T cells. At 4 weeks post-T cell infusion, recipient mice were euthanized, and bone marrow isolated for flow cytometry. Comparing the proportions of competing *TP53*-mutant and *TP53-*wildtype B-ALL, mock T cell-infused mice retained the input ratio of *TP53-*wildtype and *TP53*-mutant leukemia, suggesting no advantage of p53-deficient cells in the absence of CAR-T pressure (Fig. 1D, E). In contrast, the *TP53*-mutant proportion was significantly elevated versus *TP53-*wildtype after CAR infusion (Fig. 1D, E). To confirm this effect was not due to differences in CD19 antigen recognition and killing, we found *TP53*-mutant 697 also displayed reduced sensitivity to CD22-directed CAR-T [23] treatment compared to *TP53*-wildtype 697 in vitro (Fig. 1F), Moreover, we found no difference in CD19 antigen abundance (Fig. 1G) nor cell cycle dynamics upon CAR-T exposure, in *TP53*-wildtype versus *TP53*-mutant 697 cells (Fig. 1H), suggesting a *TP53*-mutant B-ALL-intrinsic loss of CAR-T sensitivity.

### CRISPR screen reveals lipid metabolism as a key CAR-T resistance pathway in *TP53*-mutant B-ALL

**Fig. 2:**
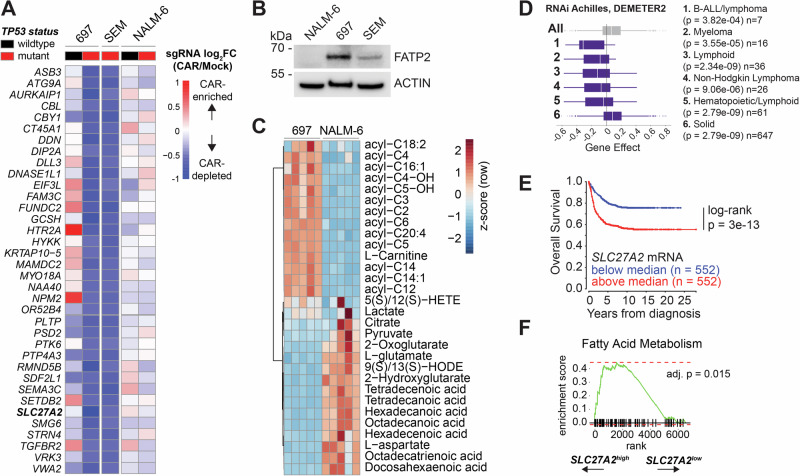
Genome-wide screen identifies fatty acid transport protein and B-ALL prognostic marker, *SLC27A2/*FATP2, as a potential CAR resistance mechanism in human B-ALL. **A** Heatmap showing log_2_fold-change of 36 gene-specific sgRNAs comparing Brunello library-transduced, isogenic B-ALL cell lines (697, SEM, NALM-6; *TP53* genotype indicated) challenged with CAR-T *versus* Mock T sgRNA representation normalized to non-targeting control sgRNAs. **B** Immunoblot analysis of FATP2 and β-Actin for NALM-6, 697 and SEM B-ALL. **C** Heatmap of differentially represented metabolites comparing 697 and NALM-6 B-ALL cell lines. Five replicates analyzed with row z-score normalization of metabolite abundance shown. **D** Gene effect plot of *SLC27A2* knockdown in human cell lines (DepMap Project). **E** Kaplan–Meier curve of pediatric B-ALL (*n* = 1104 patients) overall survival stratified by median *SLC27A2* mRNA at diagnosis (St. Jude dataset, Gu et al). **F** Gene set enrichment analysis of Fatty Acid Metabolism Hallmark dataset comparing *SLC27A2*^*high*^ and *SLC27A2*^*low*^ mRNA profiles (St. Jude dataset).

To discover mechanisms of *TP53-*mutant B-ALL resistance to CAR-T killing, we combined genome-wide CRISPR/Cas9 genetic screening approaches with CAR-T treatment ex vivo. We screened Cas9-expressing isogenic *TP53-*wildtype and *TP53-*mutant isogenic cell lines derived from 2 independent B-ALL lines (697, NALM-6), and the SEM B-ALL cell line, which harbors a heterozygous *TP53* DNA-binding domain mutation (p.R248Q) and lacks p21 induction upon Nutlin-3A treatment (Supplementary Fig. 3A). Each cell line was transduced with lentivirus encoding the Brunello genome-wide sgRNA library [24] and subjected to mock T or CD19 CAR-T cell co-culture for 72 h, at an Effector (T cell)-to-Target (B-ALL) (E:T) ratio optimized to elicit ~50% killing of each target cell line at this time point. Genes contributing to B-ALL resistance to CAR-T killing were defined as those for which corresponding sgRNAs exhibited significant depletion (log₂ fold-change <–0.5) in CAR-T–treated B-ALL cells relative to mock T-cell controls. Conversely, genes whose sgRNAs were enriched (log₂ fold-change >0.5) under CAR-T treatment were considered to enhance sensitivity to CAR-T killing (Supplementary Table 1). We saw the enrichment of NALM-6 clones carrying either *FADD* [25] or *CD19* [6] sgRNAs following CD19-directed CAR-T treatment (Supplementary Fig. 3B), consistent with previous genome-wide screening studies interrogating CAR-T resistance in NALM-6 [26]. Conversely, sgRNAs targeting *MYC*, or the CD19 repressor, *NUDT21* [27], were significantly depleted following CAR-T treatment (Supplementary Fig. 3B). To identify synthetic lethal genetic vulnerabilities that promote CAR-T killing of *TP53-*mutant B-ALL, we identified sgRNA targets depleted (log_2_fold-change reduction >0.5) in CAR T-treated *TP53-*mutant 697 and SEM B-ALL *versus* mock T-cell controls. We then removed two candidates - PTPN2 and OR8G2 - depleted in *TP53-*mutant NALM-6 screens, as NALM-6 showed no p53-dependent CAR-T responses, leaving 36 candidate genes (Fig. 2A, Supplementary Table 2). Enrichr analysis [28] of this small candidate gene set found significant associated with cellular metabolism, specifically lipid metabolism, highlighted by an enrichment of PPAR ChIP-Seq target genes (*p* = 0.001289, ChEA 2022) and genes involved in acyl-CoA biosynthesis (*p* = 0.0002828, GO Biological Processes, GO:0071616) (Supplementary Table 2).

In the absence of large clinical cohort studies profiling purified B-ALL blasts from CAR-T responders and non-responders, we analyzed publicly available RNA-sequencing data from 1104 newly diagnosed pediatric B-ALL patients from 13 clinical trials with clinical outcome data [29]. Using this dataset, we prioritized gene candidates whose high median mRNA expression associates with poorer chemotherapy outcomes, as patients with relapsed/refractory disease are indicated for CAR-T therapy. This approach yielded nine candidates, including *AURKAIP1, CBL, DDN, EIF3L*, and five genes with established roles in lipid metabolism (*FUNDC2, PLTP, PSD2, SDF2L1*, and *SLC27A2*) (Supplementary Table 2)*.* Using DepMap genome-wide screening datasets [30], we refined the selection of candidates to those essential for B-ALL cell line survival relative to non-B-ALL cell lines, where we identified a single candidate, *SLC27A2*, encoding fatty acid transporter FATP2 as a potential driver of B-ALL resistance to CAR-T therapy (Fig. 2A).

The transmembrane protein FATP2 regulates lipid homeostasis by facilitating long-chain free fatty acid transport [31]. In our screen, CD19-directed CAR-T treatment significantly depleted *SLC27A2* sgRNAs in *TP53-*mutant 697 and SEM B-ALL cell lines, with no significant sgRNA depletion in *TP53*-wildtype 697 or isogenic NALM-6 cell lines (Fig. 2A). Immunoblot analysis confirmed FATP2 expression in 697 and, to a lesser extent, SEM B-ALL, but not in NALM-6, consistent with the lack of *SLC27A2*-specific sgRNA depletion in CAR-treated NALM-6 in our CRISPR screen (Fig. 2B, Supplementary Fig. 3C). RT-qPCR analysis confirmed *SLC27A2* mRNA expression in both *TP53*-wildtype and *TP53*-mutant 697 B-ALL, but no expression in *TP53*-wildtype and *TP53*-mutant NALM-6, suggesting transcriptional control of FATP2 is not regulated by p53 in human B-ALL cell lines (Supplementary Fig. 3D). To assess whether FATP2 expression correlates with fatty acid uptake function, BODIPY uptake was measured across multiple B-ALL cell lines and quantified by flow cytometry. FATP2-expressing B-ALL cell lines (697, SEM, and additional FATP2-expressing p53-null near-haploid B-ALL, NALM-16 [32]) exhibited significantly higher BODIPY uptake than FATP2-negative NALM-6 cells (Supplementary Fig. 3E, F). In addition, whole cell metabolomics analysis confirmed a significant enrichment of FAO intermediates, acylcarnitines, present in both 697 and SEM, relative to NALM-6, consistent with an enrichment for screen hits associated with acyl-CoA biosynthesis (Fig. 2C, Supplementary Fig. 3G, Supplementary Table 3). DepMap analysis revealed *SLC27A2* knockdown preferentially reduced human B-ALL cell line survival relative to other lineages (Fig. 2D), further highlighting a functional role for FATP2 in B-ALL survival. In patients, stratification of B-ALL patient overall survival by *SLC27A2* mRNA gene expression values at diagnosis, we found *SLC27A2*^*high*^ cases associated with inferior overall survival (Fig. 2E). *SLC27A2*^*low*^ cases aligned with favorable genetic risk factors (e.g., hyperdiploid, *ETV6::RUNX1*+), whereas *SLC27A2*^*high*^ cases were linked to high-risk (*BCR::ABL1*+*, KMT2A*-rearranged, *TP53-*mutant low hypodiploid leukemia) and intermediate-risk (*TCF3::PBX1)* B-ALL subtypes (Supplementary Fig. 3H). Additional gene set enrichment analysis comparing *SLC27A2*^*low*^ and *SLC27A2*^*high*^ newly diagnosed pediatric B-ALL patients [29] revealed significant enrichment of the ‘fatty acid metabolism’ gene signature in *SLC27A2*^*high*^ cases (Fig. 2F, Supplementary Fig. 3I).

### Exogenous lipid uptake via FATP2 promotes CAR-T resistance in *TP53*-mutant human B-ALL cell lines

**Fig. 3:**
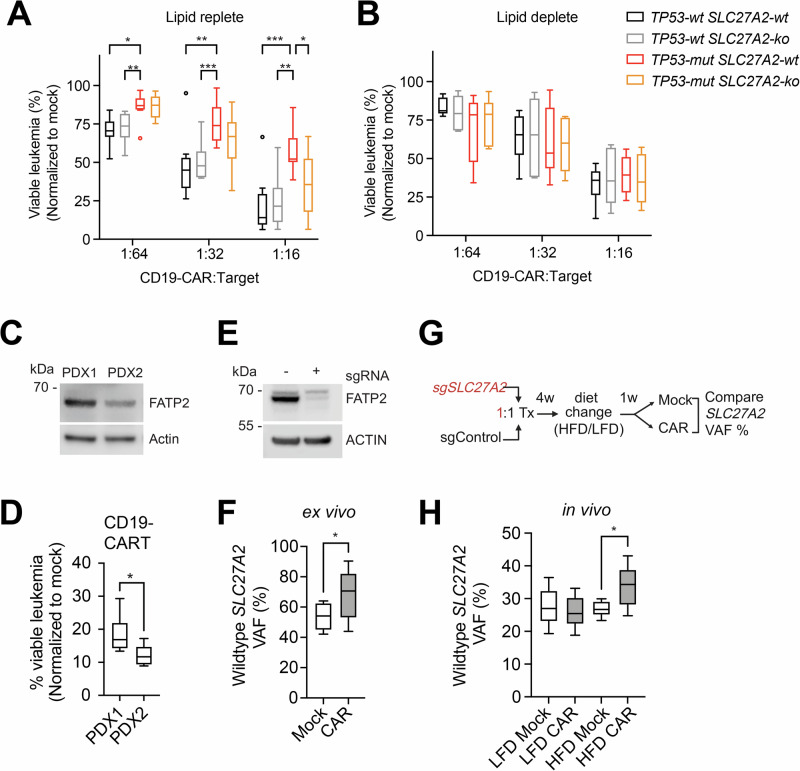
FATP2-mediated CAR-T resistance is dependent upon exogenous lipid uptake. Percentage viable leukemia (relative to Mock T cells controls) following CD19 CAR-T cell treatment of *TP53-*wildtype and *TP53*-mutant 697 B-ALL-expressing sg*ROSA* (*SLC27A2*-wildtype/wt) or sg*SLC27A2* (*SLC27A2*-knockout/ko) expression for 72 h in **A** lipid-replete or **B** lipid-deplete media. 2-way ANOVA versus *TP53*-wildtype; *SLC27A2*-wildtype, Dunnett’s multiple comparisons. *n* = 6–8 independent experiments, Tukey box-and-whisker plots. *p < 0.05, **p < 0.01, ***p < 0.005. **C** Immunoblot analysis of FATP2 and β-Actin for low hypodiploid B-ALL PDXs - PDX1 (SJHYPO009_D) and PDX2 (SJHYPO003074_D2). **D** Percentage viable leukemia (relative to Mock T cells controls) following CD19 CAR-T cell treatment of PDX1 and PDX2 B-ALL for 72-hours. E:T 1:20. *n* = 6 independent experiments. 2-way ANOVA, Tukey box-and-whisker plots. *p < 0.05. **E** Immunoblot analysis of FATP2 and β-Actin at 72 h post-electroporation with an *SLC27A2*-targeting Cas9 ribonucleoprotein complex in 697 B-ALL cells. **F** Frequency of wildtype *SLC27A2* variant alleles (measured by targeted amplicon sequencing) reads of sorted YFP+ leukemia subjected to 48-h Mock/CAR treatment ex vivo. Welch’s *t* test, *p < 0.05. *n* = 6–12 independent experiments, Tukey box-and-whisker plots. **G** Schematic of B-ALL PDX gene editing, in vivo dietary change, and T cell treatment for *SLC27A2* VAF analysis. **H** Frequency of wildtype *SLC27A2* variant alleles (measured by targeted amplicon sequencing of sorted YFP+ leukemia) reads from PDX1 B-ALL blasts from the bone marrow of mice treated with Mock/CAR treatment under low-fat diet (LFD) or high-fat diet (HFD). Welch’s *t* test, *p < 0.05. *n* = 6–9 independent experiments, Tukey box-and-whisker plots.

To test whether the transport of exogenous free fatty acids by FATP2 contributes to CAR-T cell resistance in *TP53-*mutant B-ALL, we transduced 697 *TP53*-mutant and *TP53*-wildtype 697 cell lines with lentiviral vectors expressing GFP and sgRNAs genetically inactivating *SLC27A2* or non-targeting *ROSA*26 sgRNA control. We cultured *TP53*-wildtype and *TP53-*mutant 697 cells expressing either *SLC27A2*-knockout (sg*SLC27A2*) or *SLC27A2*-wildtype (sg*ROSA*) in ‘lipid-replete” (RPMI 1640, 20% standard FBS) and ‘lipid-deplete’ media (RPMI 1640, 18% lipid-free FBS, 2% standard FBS), then treated each line with serial dilutions of Mock or CD19 CAR-T cells for 72 h (Supplementary Fig. 4A). Global lipidomic analysis of lipid-deplete and lipid-replete media confirmed a higher content of all quantified lipid classes across lipid-replete media (Supplementary Fig. 4B, Supplementary Table 4). Indeed, 218 of the 226 significantly altered lipids (>96%) were increased in lipid-replete media. In lipid-replete conditions, we found *TP53*-mutant;*SLC27A2*-wildtype cells were more resistant to CAR-T killing than *TP53*-wildtype;*SLC27A2*-wildtype cells. This resistance was dependent upon FATP2 as *TP53*-wildtype;*SLC27A2-knockout* cells were significantly more sensitive to CAR-T killing than *TP53*-wildtype;*SLC27A2*-wildtype B-ALL (Fig. 3A). In contrast, in lipid-deplete conditions, CAR-T sensitivity remained unchanged across all four isogenic B-ALL lines (Fig. 3B), suggesting exogenous lipids contribute to reduced CAR-T sensitivity in *TP53*-mutant;*SLC27A2*-wildtype B-ALL. CAR-T sensitivity varied across lipid conditions with *TP53*-mutant;*SLC27A2*-wildtype B-ALL showed significantly reduced CAR-T sensitivity with increased lipid content, an effect reversed by FATP2 loss in *TP53*-mutant;*SLC27A2-knockout* B-ALL (Supplementary Fig. 4C). In contrast, *TP53*-wildtype B-ALL exhibited increased CAR-T sensitivity with higher lipid levels, regardless of FATP2 expression (Supplementary Fig. 4C). To validate the role of FATP2 in CAR-T resistance across multiple *TP53*-mutant B-ALL cell lines, we generated Cas9-expressing NALM-16 and SEM B-ALL cells expressing GFP-sg*SLC27A2* and GFP-sg*ROSA*, followed by 72-hCD19-directed CAR-T treatment. FATP2 depletion increased the sensitivity of NALM-16 cells, and to a lesser extent, SEM B-ALL (likely due to lower baseline FATP2 expression, Fig. 2B), to CAR-T killing in vitro (Supplementary Fig. 4D, E), demonstrating FATP2 promotes CAR-T resistance across multiple B-ALL lines in a lipid-dependent manner in vitro.

### FATP2 ablation enhances CAR-T efficacy in B-ALL PDXs under high-fat, but not low-fat, conditions

We investigated the role of FATP2 in promoting CAR-T resistance using primary human *TP53*-mutated B-ALL PDX models. To assess the impact of FATP2 loss on B-ALL survival during CAR-T therapy, we utilized two CD19^+^, YFP-labeled, *TP53*-mutant low-hypodiploid PDXs – PDX1 (SJHYPO009_D, hemizygous *TP53*^R306*^, St. Jude PROPEL) and PDX2 (SJHYPO003074_D2, hemizygous *TP53*^R273C^, St. Jude PROPEL). Immunoblot analysis confirmed higher FATP2 expression in PDX1 relative to PDX2 (Fig. 3C), which correlated with lower CD19-directd CAR-T sensitivity in PDX1 relative to PDX2 ex vivo (Fig. 3D). To evaluate responses to CD19-directed CAR-T therapy in vivo, we engrafted NSG mice with YFP^+^ blasts from PDX1 and PDX2 for two weeks before infusing 100,000 Mock or CAR-T cells. CAR-T treatment significantly extended PDX1 and PDX2 disease latency compared to Mock T cell controls; however, at this dose, all CAR-treated mice succumbed to B-ALL (Supplementary Fig. 5A, B). Next, we directly tested whether FATP2 expression influenced CAR-T sensitivity. Using electroporation-based CRISPR/Cas9 editing of 697 B-ALL, we introduced pooled sgRNAs targeting two regions of *SLC27A2* exon 1 or negative control sgRNAs into the B-ALL cell line, 697. Amplicon sequencing and immunoblot analysis found extensive *SLC27A2* editing (95.29% on-target editing) and FATP2 protein loss, respectively, confirming efficient electroporation-based gene targeting of *SLC27A2* (Fig. 3E, Supplementary Fig. 5C). To assess the impact of *SLC27A2* loss on CAR-T sensitivity, we generated an admixture of *SLC27A2*-edited and *SLC27A2*-wildtype B-ALL cells (~1:1 ratio) from the FATP2-expressing PDX1 and expanded them in NSG mice. Bone marrow YFP^+^/CD19^+^ blasts were isolated from moribund recipients and subjected to ex vivo CAR-T or Mock T cell treatment on MS5 stroma for 48 h. Flow cytometry revealed a significant reduction in viable B-ALL cells and CD19 surface expression following CAR-T exposure (Supplementary Fig. 5D–F). Targeted amplicon sequencing of sorted YFP^+^ B-ALL cells demonstrated a significant increase in *SLC27A2*-wildtype variant allele frequency (VAF) in CAR-T-treated samples compared to Mock-treated controls, indicating selective enrichment of *SLC27A2*-wildtype B-ALL by CAR-T cells ex vivo (Fig. 3F).

To explore the role of exogenous fatty acids in FATP2-mediated CAR-T resistance in vivo, we transplanted the PDX1 *SLC27A2*-edited/wildtype B-ALL admixture into NSG mice, allowing tumor engraftment for four weeks. In order to increase exogenous lipid levels in tumor-bearing mice in vivo, mice were then placed on either a low-fat diet (LFD: 16 kcal% fat) or high-fat diet (HFD: 60 kcal% fat) for one week, leading to significant weight differences (Supplementary Fig. 5G, mean: LFD = 18.64 g, HFD = 20.18 g). Mice were then infused with 5 × 10⁵ Mock or CAR-T cells, YFP^+^ blasts were isolated for flow cytometry and sequencing one week later (Fig. 3G, Supplementary Fig. 5H). CAR-T treatment led to variable reductions in the total burden of B-ALL and decreased CD19 expression under both dietary conditions (Supplementary Fig. 5I, J). Under LFD conditions, there was no difference in wildtype *SLC27A2* VAF between CAR-T and Mock-treated mice (Fig. 3H). However, in HFD cohorts, CAR-T treatment increased wildtype *SLC27A2* VAF (Fig. 3H, Supplementary Fig. 5K, Supplementary Table 6), suggesting that exogenous fatty acids were necessary to promote FATP2-expressing B-ALL survival during CAR-T therapy. As a negative control, we repeated the *SLC27A2*-targeted gene-editing strategy in PDX2, which expresses lower FATP2 levels than PDX1. Following PDX2 transplantation, HFD exposure, and subsequent CAR-T or Mock treatment, we observed a reduction in bone marrow tumor burden and CD19 surface expression on YFP^+^ PDX2 blasts (Supplementary Fig. 5L, M). Targeted sequencing revealed no differences in *SLC27A2*-wildtype VAF between CAR-T and Mock-treated mice (Supplementary Fig. 5N, Supplementary Table 5), further supporting that exogenous fatty acids enhance CAR-T resistance specifically in primary FATP2-expressing B-ALL.

### FATP2-expressing *TP53*-mutant B-ALL display reduced apoptosis independent of CAR-T activity

**Fig. 4:**
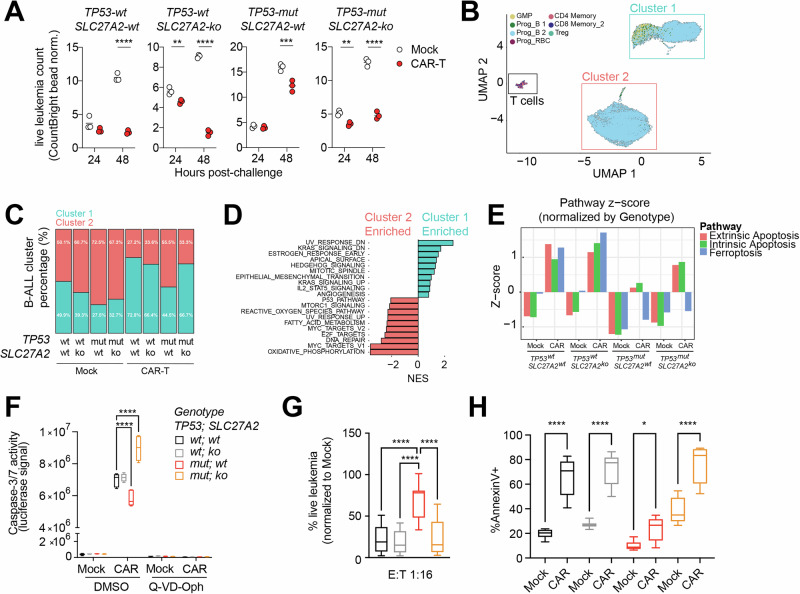
FATP2-mediated CAR-T resistance is associated with reduced apoptosis of *TP53*-mutant B-ALL. **A** Histogram of live leukemia cells (normalized to fixed number of CountBright beads) for 697 B-ALL cell lines exposed to CAR-T (1:16 E:T ratio) at 24 and 48 h. 2-way ANOVA versus Mock, *n* = 3 replicates, mean shown. **p < 0.01, ***p < 0.005, ****p < 0.0001. **B** UMAP representation of single-cell RNA-sequencing data from all four isogenic 697 B-ALL cell lines at 48-hours post-Mock or CAR-T exposure (1:16 E:T ratio). Manually defined cell clusters are shown, as well as Seurat cell type annotations. **C** Histogram of the percentage of B-ALL cells present in Cluster 1 and Cluster 2 for each genotype with T cell treatment shown. **D** Gene set enrichment analysis of differentially expressed genes when comparing B-ALL Cluster 1 and Cluster 2. Top 10 enriched pathways for either cluster are shown. **E** Histogram of Seurat Module Scores for isogenic 697 B-ALL cell lines following Mock or CAR-T exposure. Z-score normalization of each pathway across all samples shown. **F** Histogram of caspase-3/7 activity for isogenic 697 B-ALL cell lines at 48-hours post-Mock or CAR-T exposure (1:16 E:T ratio). Raw chemiluminescence signal shown. 2-way ANOVA relative to *TP53*-wildtype *SLC27A2*-wildtype shown. *n* = 6 independent experiments, Tukey box-and-whisker plots. Histogram of matched (**G**) percentage live leukemia cells (normalized to Mock T cell control) and (**H**) percentage AnnexinV+ cells for 697 B-ALL cell lines exposed to CAR-T (1:16 E:T ratio) 72 h. **G** 2-way ANOVA shown, *n* = 12 replicates, three independent experiments, Tukey box-and-whisker plots. ****p < 0.0001. **H** 1-way ANOVA comparing CAR versus Mock, *n* = 12 replicates, three independent experiments, Tukey box-and-whisker plots. **p < 0.01, ****p < 0.0001.

To investigate the kinetics of FATP2-mediated CAR-T resistance, we challenged all four isogenic 697 B-ALL lines with CAR-T cells for 24 and 48 h, followed by flow cytometry analysis. After 24 h of co-culture, we observed a subtle decrease in B-ALL survival in both *TP53*-wildtype;*SLC27A2-knockout* and *TP53*-mutant;*SLC27A2-knockout* lines, whereas *TP53*-wildtype;*SLC27A2*-wildtype and *TP53*-mutant;*SLC27A2*-wildtype lines showed no significant changes (Fig. 4A). By 48 h, we observed significant CAR-T-mediated killing across all B-ALL groups, however, CAR-T killing of *TP53*-mutant;*SLC27A2*-wildtype cells were markedly attenuated (Fig. 4A). To investigate the transcriptional basis of differential CAR-T responses at 48 h, we performed single-cell RNA sequencing (scRNA-seq) on B-ALL/CAR-T co-cultures. Using Seurat cell type annotation and manual clustering, we identified two distinct B-ALL clusters (Cluster 1 and Cluster 2), both primarily composed of ‘Prog_B2’ cells, along with a separate T cell cluster (Fig. 4B). Next, we quantified changes in the proportion of B-ALL cells within Cluster 1 and Cluster 2 for each isogenic B-ALL line following CAR-T treatment, relative to Mock T cell controls. In Mock T cell-treated condition, *TP53*-mutant B-ALL displayed a higher proportion of Cluster 2 relative to Cluster 1 when compared to *TP53*-wildtype B-ALL. Upon CAR-T treatment, ‘CAR-sensitive’ lines (*TP53*-wildtype;*SLC27A2*-wildtype, *TP53*-wildtype;*SLC27A2-knockout*, and *TP53*-mutant;*SLC27A2-knockout*) resulted in an increased reduction in the proportion of Cluster 2 B-ALL cells when compared *TP53*-mutant;*SLC27A2*-wildtype B-ALL cells that exhibited an attenuated reduction in Cluster 2 (Fig. 4C). Gene set enrichment analysis revealed significant transcriptional differences between the B-ALL clusters, with Cluster 2 showing enrichment in oxidative phosphorylation, fatty acid metabolism, and p53 signaling pathway signatures (Fig. 4D, Supplementary Fig. 6A). To identify survival pathways underpinning differences in Cluster 2 reduction following CAR-T across B-ALL lines, we performed gene set enrichment analysis of intrinsic apoptosis, extrinsic apoptosis and ferroptosis gene expression programs in Mock and CAR-treated Cluster 2 B-ALL cells (Fig. 4E). Notably, independent of FATP2 expression, CAR-T treatment *TP53*-wildtype B-ALL showed a significant induction of ferroptosis gene signatures relative to *TP53*-mutant B-ALL (Fig. 4E). However, CAR-T treatment of the three CAR-sensitive lines resulted in a significant increase in both intrinsic and extrinsic apoptosis gene signatures, whereas induction of these pathways was significantly lower in *TP53*-mutant;*SLC27A2*-wildtype cells (Fig. 4E). To functionally test this observation, we performed CAR-T treatment of isogenic B-ALL cell lines in the presence or absence of pan-Caspase inhibitor, Q-VD-Oph/QVD, followed by caspase-3/7 activity measurement. Here, we observed elevated caspase-3/7 activity in CAR-T treated B-ALL lines relative to Mock-treated controls, which was readily reversed by QVD treatment. Notably, the induction of caspase-3/7 was significantly reduced in *TP53*-mutant;*SLC27A2*-wildtype relative to CAR-sensitive lines (Fig. 4F). To confirm these findings, we performed Annexin-V staining of CAR-T treated B-ALL lines relative to Mock-treated controls (Fig. 4G, H, Supplementary Fig. 6B), further demonstrating that the combination of *TP53* loss and FATP2 expression reduced induction of apoptosis following CAR-T treatment. Extending on our CAR-T response findings, we then investigated FATP2-dependent responses to conventional ALL chemotherapy (specifically L-Asparaginase, Cytarabine/Ara-C, Vincristine, and Dexamethasone) in isogenic 697 and NALM-6 cells. We found that dexamethasone responses were independent of p53 and/or FATP2 expression in both NALM-6 and 697 (Supplementary Fig. 6C). Vincristine responses were similarly attenuated in *TP53*-mutant 697 and NALM-6 cells, independent of FATP2 expression (Supplementary Fig. 6C). Interestingly, *TP53*-mutant;*SLC27A2*-wildtype 697—but not *TP53*-mutant;*SLC27A2*-wildtype NALM-6—showed a pattern of resistance similar to their CAR-T responses when challenged with the anti-metabolite Ara-C and L-Asparaginase (Supplementary Fig. 6C), suggesting that FATP2 mediates a specific mechanism of resistance in *TP53*-mutant 697 B-ALL which does not extend to all therapeutics.

To assess whether differences in CAR-T responses were linked to altered T cell function, we analyzed scRNA-seq data from the T cell cluster (Fig. 4B). We observed no significant differences in T cell subset identity (based on Seurat cell type annotation) between Mock and CAR-T treatments, nor in *GZMB* mRNA expression in response to any of the four isogenic B-ALL lines (Supplementary Fig. 7A, B). Flow cytometry analysis of the T cell activation marker CD69 on Mock and CAR-T cells following 72 h of co-culture with all four isogenic B-ALL lines under lipid-replete and lipid-deplete conditions revealed no significant differences in the proportion of CD69 + CAR-T cells (Supplementary Fig. 7C, D). We then tested whether changes in B-ALL CD19 antigen levels upon CAR-T exposure may account for differential CAR responsiveness, however, we did not observe any differences baseline surface CD19 expression nor CD19 antigen reduction following CAR-T exposure across all four cell lines (Supplementary Fig. 7E). To assess whether FATP2 activity alters mechanical forces and affinity of immunological synapses formed between CAR-T and B-ALL [33], we performed atomic force microscopy (AFM) on isogenic 697 *TP53*-wildtype and *TP53*-mutant B-ALL lines with or without FATP2 expression, and imaging flow cytometry analysis of CAR-T:B-ALL conjugates to evaluate synapse frequency and size (Supplementary Fig. 7F–I). These analyses revealed no significant differences in B-ALL membrane stiffness (Young’s modulus) across isogenic lines. Similarly, CAR-T/B-ALL conjugate frequency (CAR:GFP+ synapses) and synapse size (phalloidin intensity at synaptic area) did not differ based on the genetic background of the B-ALL cells (Supplementary Fig. 7F–I), thus suggesting a FATP2-mediated intracellular mechanism of CAR-T resistance in *TP53*-mutant B-ALL.

### FATP2 promotes CAR-T resistance by enhancing FAO via lipid uptake

**Fig. 5:**
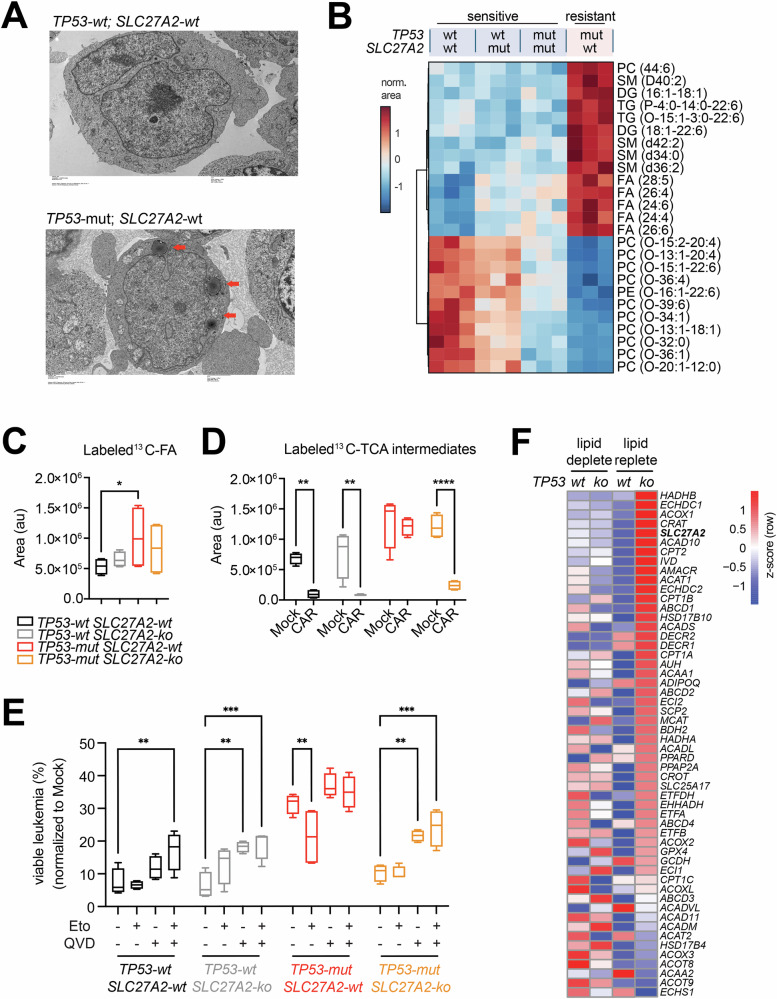
Elevated FAO promotes FATP2-mediated CAR-T resistance. **A** Representative electron microscopy of *TP53*-wildtype and *TP53*-mutant 697 B-ALL with lipid droplets indicated (red arrows). **B** Top 25 differentially quantified lipids (by ANOVA) across Mock-treated isogenic 697 B-ALL lines, as expressed by normalized area with genotypes shown. Histogram of ^13^C-labeled metabolite abundance (measured by Area, arbitrary units) for (**C**) intracellular ^13^C-LCFA (palmitate, oleate, linoleate) in Mock-treated B-ALL, and (**D**) ^13^C-labeled TCA intermediates (sum of citrate, a-KG, succinate, fumarate and malate) for CAR- versus Mock-treated isogenic 697 B-ALL lines. 2-way ANOVA. *n* = 4 independent experiments, Tukey box-and-whisker plots. **E** Live leukemia count (normalized to Mock/DMSO control) following Mock or CD19 CAR-T cell treatment (1:16 E:T) of *TP53-*wildtype and *TP53*-mutant 697 B-ALL-expressing sg*ROSA* (*SLC27A2*-wildtype) or sg*SLC27A2* (*SLC27A2-knockout*) expression for 72 h with co-treatment of DMSO control and/or CPT1 inhibitor Etomoxir (5 µM). 2-way ANOVA versus *TP53-*wildtype *SLC27A2*-wildtypes. *n* = 4 replicates. Tukey box-and-whisker plots. **F** Heatmap showing log_2_fold-change of FAO-specific sgRNAs (Gene Ontology:0006635) comparing Human Metabolic Genes CRISPRa sgRNA library-transduced, isogenic dCas9-VPR 697 *TP53*-wildtype and *TP53*-mutant B-ALL cell lines challenged with CAR-T *versus* Mock T under lipid replete and deplete culture conditions, with sgRNA representation normalized to non-targeting control sgRNAs.

Based on the lipid uptake function of FATP2, we performed neutral lipid staining of B-ALL cell lines which showed increased lipid droplet formation in *TP53*-mutant;*SLC27A2*-wildtype B-ALL cells relative to both *TP53*-wildtype;*SLC27A2*-wildtype and *TP53*-mutant;*SLC27A2-knockout* counterparts (Supplementary Fig. 8A), which was further validated through electron microscopy of *TP53*-mutant;*SLC27A2*-wildtype versus *TP53*-wildtype *SLC27A2*-wildtype cells (Fig. 5A, Supplementary Fig. 8B). Lipid droplet biogenesis involves glycolytic product, glycerol-3-phosphate and fatty acids providing essential substrates to produce neutral lipids for storage, such as triglycerides (TG) [34]. Using metabolic flux assays, we found that CAR-resistant *TP53*-mutant;*SLC27A2*-wildtype B-ALL displayed elevated glycolysis and non-glycolytic acidification, relative to CAR-sensitive lines (Supplementary Fig. 8C, D). To investigate how specific lipids were utilized by B-ALL in the presence or absence of CAR-T therapy, we performed global lipidomic analysis on sorted GFP^+^ B-ALL cells from all four isogenic 697 B-ALL lines following challenge with Mock or CAR-T cells under lipid-replete conditions. In the absence of CAR-T pressure, we observed a significant increase in diglycerides (DG), TGs, and polyunsaturated fatty acid (FA) levels in CAR-resistant B-ALL relative to the three CAR-sensitive B-ALL lines (Fig. 5B, Supplementary Table 6), consistent with increased lipid droplet formation in CAR-resistant B-ALL cells. In addition, *TP53*-wildtype B-ALL displayed a higher phosphatidylcholine (PC) content when compared to *TP53*-mutant B-ALL, consistent with a role for p53 in suppression of lipid droplet coalescence [35]. To assess dynamic changes in lipid content in response to CAR-T pressure, we performed a differential lipid abundance analysis comparing Mock-treated and CAR-T-treated CAR-resistant and CAR-sensitive B-ALL. CAR-sensitive B-ALL exhibited a significant reduction in global lipid content after CAR-T therapy, whereas no such reduction was observed in CAR-resistant B-ALL (Supplementary Fig. 8E). Further analysis identified phospholipids as the primary lipid species reduced in CAR-sensitive B-ALL, following CAR-T therapy when compared to CAR-resistant B-ALL cells (Supplementary Fig. 8F). To further dissect the mechanistic basis of lipid utilization to promote survival under CAR-T pressure, we cultured 697 CAR-resistant and CAR-sensitive B-ALL lines in media containing a mixture of ^13^C-labeled long chain fatty acids (LCFAs), specifically, linoleic (polyunsaturated), oleic (monounsaturated) and palmitic (saturated) acids. To investigate the relative uptake of LCFAs across all four cell lines, we quantified both intracellular and culture media isotopic ^13^C-LCFAs abundance following 72-hours of culture (Fig. 5C, Supplementary Fig. 8G, Supplementary Table 7). Notably, CAR-resistant *TP53*-mutant;*SLC27A2*-wildtype B-ALL showed an elevated intracellular ^13^C-LCFA when compared to CAR sensitive lines. In addition, the opposite effect was observed for *TP53*-mutant;*SLC27A2-knockout* lines, which showed a higher abundance of ^13^C-LCFA in media compared to all other B-ALL cell lines, suggesting that *TP53*-mutant CAR-resistant cells display elevated FATP2-dependent LCFA uptake compared to sensitive B-ALL lines (Fig. 5C, Supplementary Fig. 8G). Isogenic 697 cell lines cultured in ^13^C-LCFA media were subsequently challenged with Mock or CD19-CAR-T cells for 72-hours, followed by differential analysis of ^13^C-labeled metabolites. For CAR-sensitive B-ALL, we found a dramatic decrease in ^13^C-labeled TCA cycle intermediates following CAR-T exposure, which was not observed in CAR-resistant B-ALL (Fig. 5D). Stored neutral lipids (e.g., TGs) may undergo lipase-mediated lipolysis to liberate FFAs for CPT1-mediated transport of FFAs into the mitochondria providing FAO substrates required for energy production. To test whether this stepwise process contributes to CAR resistance, we treated isogenic B-ALL cells with the lipase inhibitor Lalistat-1 (LAL1i) during co-culture with Mock or CAR-T cells. Notably, LAL1i treatment was performed at concentrations capable of inhibiting both lysosomal and neutral lipases [36]. We found that LAL1i treatment reduced B-ALL viability as single agents (Supplementary Fig. 9A). In *TP53*-wildtype B-ALL, CAR-T treatment responses were unaffected by the addition of LAL1i, whereas *TP53*-mutant B-ALL exhibited significantly enhanced CAR-T sensitivity in the presence LAL1i; however, these effects are unlikely to be solely FATP2-dependent, as LAL1i also enhanced CAR-T sensitivity in *TP53*-mutant;*SLC27A2-knockout* B-ALL (Supplementary Fig. 9A). In addition to targeting stored lipids, we assessed whether CPT1-mediated FAO impacts CAR-T-mediated apoptosis. To do this, we treated isogenic B-ALL cells with the CPT1 inhibitor Etomoxir and/or the pan-caspase inhibitor QVD during Mock or CAR-T co-culture. In CAR-sensitive lines—*TP53*-wildtype;*SLC27A2*-wildtype, *TP53*-wildtype;*SLC27A2*-knockout, and *TP53*-mutant;*SLC27A2*-knockout—CAR-T-induced apoptosis was not enhanced by Etomoxir and was readily reversed by QVD (Fig. 5E). Conversely, CAR-T-induced killing of *TP53*-mutant;*SLC27A2*-wildtype B-ALL was increased in the presence of Etomoxir and reversed by QVD, suggesting that blocking CPT1-mediated FAO improves CAR-T sensitivity to caspase-dependent apoptosis in *TP53*-mutant;*SLC27A2*-wildtype B-ALL (Fig. 5E). To understand the broader role of FAO in promoting CAR-T resistance, we tested the importance of FAO in human *TP53*-mutant AML cell lines. To achieve this, we derived isogenic *TP53*-wildtype and *TP53*-knockout lines from the MOLM-13, MOLM-14, and OCI-AML3 AML cell lines. Each line was then challenged with CD33-directed CAR-T in the presence or absence of Etomoxir at serial doses for 72 h. *TP53*-knockout AML lines were less sensitive than *TP53*-wildtype AML lines to CAR-T killing in both MOLM-13 and MOLM-14; however, we did not observe this effect in OCI-AML3, although the mechanism underlying this remains unclear (Supplementary Fig. 9B). We then assessed whether Etomoxir enhanced CAR-T sensitivity in *TP53*-knockout AML lines. Here, Etomoxir did not promote CAR-T killing in *TP53*-wildtype or *TP53*-mutant AML, suggesting that, unlike B-ALL lines tested, FAO is not a driver of CAR-T resistance in *TP53*-mutant MOLM-13 and MOLM-14 (Supplementary Fig. 9B).

To validate FAO as a potential driver of B-ALL CAR-T resistance in lipid-rich conditions, we generated *TP53*-mutant (*TP53* frameshift knockout) and *TP53*-wildtype 697 B-ALL stably expressing dCas9-VPR, allowing for gene-specific CRISPR activation (CRISPRa). *TP53*-mutant dCas9-VPR 697 B-ALL showed reduced CAR-T sensitivity relative to *TP53*-wildtype dCas9-VPR 697 B-ALL (Supplementary Fig. 9C). We then tested dCas9-VPR activity through lentiviral ectopic co-expression of *CD22*-targeting sgRNAs (or sgROSA controls) and mCherry. Here, sgCD22+ dCas9-VPR + 697 B-ALL showed elevated surface CD22 expression relative to sgROSA+ dCas9-VPR + 697 B-ALL, as well as non-transduced mCherry- B-ALL cells (Supplementary Fig. 9D). Each cell line was transduced with lentivirus encoding a human metabolism-focused CRISPRa sgRNA library [37] and co-expression of a puromycin-resistance cassette (>500-fold sgRNA transduction coverage at multiplicity of infection [MOI] <1) for two days. After puromycin selection of sgRNA+ cells for 3 days, cells were split into lipid replete or lipid deplete media, then subjected to mock T or CD19 CAR-T cell co-culture for 72 h, at an Effector (T cell)-to-Target (B-ALL) (E:T) ratio optimized to elicit ~50% target killing at this time point. Following co-culture, we isolated genomic DNA to PCR amplify sgRNAs for deep sequencing. We calculated the average sgRNAs/gene, normalized counts to control sgRNAs, and compared sgRNA representation in Mock- versus CAR-T-treated conditions. Metabolism genes contributing to CAR-T resistance in human B-ALL were defined as those for which corresponding sgRNAs exhibited significant enrichment (log₂ fold-change >0.5) in CAR-T–treated B-ALL cells relative to mock T-cell controls. Quantification of sgRNAs targeting genes involved in FAO (Gene Ontology:0006635) demonstrated a significant enrichment of FAO genes (e.g., *SLC27A2, CPT1A, CPT1B, ACOX1*) following CAR-T treatment of *TP53*-mutant B-ALL compared to *TP53* wildtype B-ALL. Enrichment of FAO genes was dependent upon access to exogenous lipids, as it was not observed in *TP53*-mutant B-ALL cultured in lipid deplete conditions (Fig. 5F, Supplementary Fig. 9E). This aligns with our CRISPR knockout screening data, suggesting elevated FAO promotes CAR-T resistance in *TP53*-mutant B-ALL when exogenous lipids are available.

## Discussion

Beyond antigen modulation, the B-ALL-intrinsic mechanisms underlying resistance to CAR-T therapy remain poorly understood. Multiple studies have performed genome-wide CRISPR screening in NALM-6 B-ALL cells treated with CD19-directed CAR-T cells or bi-specific T cell engager, blinatumomab, identifying key regulators of CD19-directed T cell therapy efficacy, including activation of the proapoptotic death receptor pathway [25], intrinsic apoptosis via NOXA [26], and CD58-mediated T-cell interactions [38]. Our study identifies fatty acid metabolism following FATP2-mediated lipid uptake alters the sensitivity of FATP2-expressing B-ALL blasts to CAR-T cell therapy. While we do not observe differences in short-term CAR-T cell synapse formation or activation in our co-culture studies, these assays did not assess dynamic differences in CAR synapse integrity and duration, which have been shown to be crucial for CAR-T cytolytic activity [39, 40]. In addition, recent studies suggest that fatty acid uptake significantly alters tumor-associated T-cell cytotoxicity [41, 42] and may also promote an immunosuppressive immune microenvironment in a FATP2-dependent manner in vivo [42]; therefore, we cannot discount the possibility that differences in lipid content may affect both direct and indirect factors that modulate CAR-T function.

FAO has emerged as a potential therapeutic target across multiple hematological and solid malignancies, including CAR-T [43] and chemotherapy-resistant disease [44, 45]. For example, etomoxir-mediated inhibition of FAO enhances leukemia cell sensitivity to BH3 mimetic–induced apoptosis [46], while T cell–derived interferon-γ can upregulate FAO and contribute to CAR-T resistance in solid tumor models [43]. In our study, *TP53*-mutant B-ALL resistance to CAR-T could be reversed by pharmacological inhibition of lipolysis and FAO. Together with our finding that *TP53*-mutant B-ALL exhibits FATP2-dependent resistance to select conventional chemotherapies (e.g., Ara-C, L-asparaginase), these results highlight the need for future mechanistic studies to define the convergent pathways through which FATP2 promotes leukemic cell survival across distinct therapies.

Our findings also suggest a potential interaction between *TP53* mutation status and fatty acid metabolism in mediating CAR-T resistance; however, the precise regulatory relationship between p53 and FATP2 remains unclear. Our observations align with prior reports demonstrating that p53 loss promotes lipid droplet accumulation, potentially due to defects in Kennedy pathway activation [35]. Emerging evidence suggests that p53 may exert context-dependent effects on lipid metabolism [47], further supporting the need to incorporate a broader range of B-ALL subtypes across human cell lines and PDX models to delineate the specific genetic and cell-of-origin determinants that underlie p53-dependent CAR-T and chemotherapy resistance.

Overall, our findings highlight a previously unappreciated link between lipid metabolism and CAR-T resistance in B-ALL; however, the lack of publicly available genomic and transcriptomic profiling of purified leukemic cells from large CAR-T clinical trial cohorts limits the full extrapolation of these findings to experimental findings and general clinical implications. However, these findings suggest that evaluating lipid uptake and downstream FAO as correlates of clinical outcomes in future CAR-T cell studies is warranted to better understand the determinants of CAR-T cell efficacy and resistance in B-ALL patients.

## Methods

### Sex as a biological variable

Equal numbers of NSG male and female mice were used in in vivo experiments.

### Statistics

Data were analyzed using Prism, version 10.4.1 (GraphPad Software). Individual statistical analyses and methodology are detailed in the Fig. legends for Fig.s presenting statistical comparisons.

### Study approval

Mice were housed in individually ventilated cages and provided autoclaved food and water at the University of Colorado Anschutz Medical Campus Animal Facility. All procedures conducted under approved IACUC protocols in accordance with institutional and national guidelines (PI: Witkowski, Protocol #01188).

### Cell line and PDX culture

Human B-ALL cell lines Reh (RRID:CVCL_1650), 697 (RRID:CVCL_0079), NALM-6 (RRID:CVCL_0092), and NALM-16 (RRID:CVCL_1834) were cultured in lipid-replete RPMI-1640 (20% FBS, 55 μM β-mercaptoethanol, penicillin/streptomycin; Gibco #15140122) or lipid-deplete RPMI-1640 (2% FBS, 18% lipid-depleted FBS [Captivate Bio FBS162LD], 55 μM β-mercaptoethanol, penicillin/streptomycin). Human AML cell lines MOLM-13, MOLM-14 and OCI-AML3 were cultured in lipid-replete RPMI-1640. HEK293T cells (RRID:CVCL_0045; ATCC CRL-1573) and MS-5 stroma (RRID:CVCL_2128) were maintained in DMEM with 10% FBS and penicillin/streptomycin. All tissue-culture reagents were from Gibco. Reh, 697, and NALM-6 were provided by Dr. William Carroll (NYU); NALM-16 by Dr. Ernesto Flores-Diaz (UCSF); MS-5 by Dr. James DeGregori (CU Anschutz). MOLM-13, MOLM-14 and OCI-AML3 were provided by the Dr. Iannis Aifantis (NYU).

PDXs were cultured on retronectin-coated plates (10 mg/mL, Takara Bio) in X-VIVO 10 supplemented with 1% BSA, 1% L-glutamine, and human cytokines: FLT3-L (100 ng/mL), G-CSF (10 ng/mL), IL-6 (10 ng/mL), SCF (100 ng/mL), TPO (15 ng/mL), and IL-7 (50 ng/mL). All cultures were maintained at 37 °C, 5% CO₂. Nutlin-3a (MedChemExpress HY-10029) was used as described with DMSO vehicle control.

Cell line authentication was performed routinely using GlobalFiler® STR profiling (24 loci plus Amelogenin) on the ABI 3500XL Genetic Analyzer, with data analyzed in GeneMapper® v6.0 and appropriate allelic ladders and positive/negative controls. Mycoplasma testing was performed using the Lonza MycoAlert assay, with cultures confirmed negative prior to banking and maintained for up to 30 days after confirmed negativity.

### Gene editing of human B-ALL cell lines and PDXs

Reh, 697, NALM-6, NALM-16, MOLM-13, MOLM-14 and OCI-AML3 Cas9-expressing lines were generated by lentiviral transduction with Lenti-Cas9-2A-blast (Addgene #73310). dCas9-VPR 697 lines were produced using lentiviral dCas9-VPR (Horizon Discovery CAS11916). Lentivirus was generated in HEK293T cells using PEI and a 4:2:3 ratio of sgRNA/Cas9 : pVSVG (Addgene 138479) : pPax2 (Addgene 12260) in Opti-MEM. Spin infections were performed at 800 × *g* for 30 min at room temperature with polybrene.

Individual gene targeting was achieved using pLRG-based vectors (GFP, TagBFP2, or mCherry variants) expressing sgRNAs targeting *ROSA26, TP53, CD22 or SLC27A2*. To generate *TP53* frameshift mutant B-ALL and AML, cells were transduced with pLRG constructs (LRCherry2.1, Addgene #108099; LRG2.1-TagBFP2, Addgene #124773) expressing sg*TP53* (mCherry⁺) or non-targeting sgROSA (BFP⁺), followed by single-cell cloning and *TP53* amplicon sequencing. For FATP2-deficient lines, mCherry⁺ *TP53*-mutant or BFP⁺ *TP53*-wildtype isogenic lines were further transduced with GFP-expressing LRG2.1 vectors (Addgene #108098) encoding sgROSA or sg*SLC27A2*, and BFP⁺GFP⁺ or mCherry⁺GFP⁺ cells were sorted. All sgRNA sequences are provided in Supplementary Table 9.

To model *TP53* c.734G>A (G245D), we used an electroporation-based HDR approach (IDT). *TP53*-targeting crRNA (100 µM) was annealed with tracrRNA (100 µM), complexed with Cas9 (62 µM), incubated 20 min, and combined with donor oligos and electroporation enhancer (100 µM each). Cas9-expressing B-ALL lines were resuspended in Lonza SF buffer and electroporated (CA-137 program, 4D Nucleofector). Clones were isolated and validated by targeted amplicon sequencing (QuintaraBio).

### Amplicon sequencing

Genomic DNA (>1 ng) was subjected to PCR amplification using Q5 HotStart High-Fidelity Master Mix (New England Biolabs, Cat# M0494), 10 µM forward primer, 10 µM reverse primer using the following PCR conditions: initial denaturation at 98 °C for 1 min, 35 cycles of amplification (98 °C for 10 s, 66 °C for 30 s, 72 °C for 30 s), followed by final extension at 72 °C for 2 min and 12 °C hold. Amplified DNA was purified using the AMPureXP Cleanup Reagent (Beckman Coulter, Cat# A63880) followed by for Amplicon sequencing (QuintaraBio AmpExpress). Oligo sequences and PCR conditions for Amplicon sequencing (QuinataraBio AmpExpress) are detailed in Supplementary Table 9.

### Generation of CAR T-cells and CAR-T killing assays

Lentivirus encoding CD19, CD22 and CD33 CAR transgenes—based on the Tisagenlecleucel CD19 sequence, a clinically-tested CD22-BB design [23] and published CD33 preclinical construct [48]—was generated as previously described [23, 49, 50] by transient transfection of Lenti-X 293T cells (Takara Bio) with transfer and packaging plasmids (pRSV-Rev, pMDLg/pRRe, pMD2.G; Addgene 12253, 12251, 12259) using Lipofectamine 3000 in Opti-MEM. Media was replaced after 6 h, and viral supernatant was collected at 24 and 56 h, cleared (3000 × *g*, 10 min), aliquoted, and stored at –80 °C.

Human peripheral blood T cells were thawed at 1 × 10⁶ cells/mL and activated with CD3/CD28 Dynabeads (3:1 bead:cell) in AIM-V medium containing 5% heat-inactivated FBS, penicillin/streptomycin (100 U/mL each), GlutaMax, 10 mM HEPES, and 40 IU/mL IL-2. T cells were transduced at MOI 2–10 via spinfection (1000 × *g*, 2 h, 32 °C) with 40 IU/mL IL-2 and 10 μg/mL protamine sulfate, incubated overnight (37 °C, 10% CO₂), and bead-depleted the next day. CAR T cells were expanded at 0.5 × 10⁶/mL in 100 IU/mL IL-2 for 4 days, and CAR expression was confirmed by flow cytometry using PE-conjugated Whitlow/218 linker antibody (CST E3U7Q). Cells were cryopreserved 8 days post-activation and thawed one day before use.

CAR T cells were co-cultured with B-ALL lines (BFP⁺GFP⁺ or mCherry⁺GFP⁺) or GFP⁺ PDXs at the indicated effector:target ratios in lipid-replete or, where specified, lipid-deplete media with 4 U/mL IL-2 for 48–72 h. PDX assays were performed on MS-5 stroma. Target cells were seeded at a uniform density of 20,000 cells per 0.32 cm². Mock T cells were included at the highest E:T ratio (1:8) to control for allogeneic T cell activity. All CAR-T experiments used a minimum of two independent human T-cell donors.

### B-ALL in vivo CAR-T therapy

Non-irradiated 4–8-week-old NSG mice (male and female) were intravenously engrafted with 1 × 10⁵ B-ALL cells (697 cell lines or luciferase⁺GFP⁺ PDXs). For diet-switch studies, mice were placed on either a high-fat diet (HFD; 60% kcal fat, D12492i) or low-fat diet (LFD; 10% kcal fat, D12450Ji), matched for sucrose (Research Diets, Inc.). Mice were injected with 5 × 10⁵ Mock or CAR-T cells without pre-conditioning, given p53-dependent apoptosis induced by irradiation. CAR-T dosing was based on CAR⁺ cell number, and Mock doses were matched to total T-cell number. Mice were monitored for xenogeneic GVHD (skin/eye redness, weight loss, fur loss). At pre-defined endpoints, mice were euthanized, and bone marrow was isolated from femurs and tibias, processed into single-cell suspensions, RBC-lysed, stained, and analyzed by flow cytometry or used for sorting of B-ALL blasts.

### Flow cytometry

Cells were centrifuged at 400 × *g* for 5 min at 4 °C, supernatant removed, and pellets resuspended in PBS with 2% FCS. After a second identical wash, single-cell suspensions were incubated in PBS/2% FCS with Human TruStain FcX (BioLegend 422301) for 10 min on ice, washed, then stained with antibody cocktails in PBS/2% FCS for 30 min on ice, followed by two washes. Final samples were resuspended in PBS/2% FCS containing CountBright™ Absolute Counting Beads (5% v/v; Invitrogen C36950) and a viability dye. Antibody panels are listed in Supplementary Table 10.

Cell sorting was performed on a Sony MA900 or Astrios EQ 70; flow cytometry was acquired on a BD Fortessa and analyzed with FlowJo v10. Gating included FSC/SSC, doublet exclusion, viability gating (e.g., DAPI^–^ or ViaProbe780^–^), and identification of the target population (e.g., GFP⁺ sgRNA-expressing B-ALL). Beads were gated by FSC/SSC and confirmed by fluorescence; normalized live cell counts were calculated as target-cell events divided by bead counts.

For BFP/*TP53*-wildtype and mCherry/*TP53*-mutant competition assays, co-cultures were stained with viability dye and analyzed to determine mCherry/*TP53*-mutant:BFP/ *TP53*-wildtype ratios (Supplementary Fig. 2A). For *TP53*^G245D/+^ vs BFP/*TP53*^⁺/⁺^ assays (*TP53*^G245D/+^ lacking a fluorescent reporter), co-cultures were stained with CD3-conjugated antibodies and viability dye to quantify *TP53*^G245D/+^ (CD3^–^BFP^–^):*TP53*-wildtype (CD3^–^BFP⁺) ratios.

### Intracellular DAPI and BODIPY assays

DAPI DNA content assays were performed using DAPI counterstaining following BioLegend Cyto-Fast Fix/Perm Buffer set (Cat# 426803). For BODIPY uptake assays, we serum-starved B-ALL in RPMI 1640 media for 1 h; then added 1 µM long-chain BODIPY-C_13_ (BODIPY™ 493/503, Invitrogen) for 20 min, with BODIPY uptake quantified by flow cytometry.

### Cell titer Glo, Caspase-3/7 activity and Annexin V apoptosis assays

B-ALL cell lines were maintained in lipid-replete medium at 37 °C with 5% CO₂. For viability assays, 20,000 cells were plated in triplicate in 96-well plates (200 µL/well) and cultured for 72 h under the indicated treatments. Cell viability was measured using CellTiter-Glo® (Promega) by adding an equal volume of reagent, shaking for 2 min, incubating 10 min at room temperature, and recording luminescence; background from medium-only wells was subtracted. Caspase activity in CAR-T co-cultures was quantified using the Caspase-Glo® 3/7 Assay (Promega G8090). An equal volume of reagent was added to each well, plates were incubated for 60 min, and luminescence was measured with background subtraction. For Annexin-V staining, cells were stained with APC-conjugated Annexin V (BioLegend, Cat# 640920) in Annexin V Binding Buffer (BioLegend, Cat# 422201) according to the manufacturer’s instructions, with samples run on the BD Fortessa then analyzed using the FlowJo v10.

### Brunello CRISPR screen and metabolism-focused screen

Genome-wide CRISPR knockout and metabolism-focused CRISPRa screens were performed using the Brunello library (Addgene #73179; 19,114 genes, 77,441 sgRNAs) and the Human Metabolic Gene CRISPRa library (Addgene #187080; 2,989 genes, 32,460 sgRNAs, including 49 non-targeting controls). sgRNAs were cloned following Zhang laboratory protocols and subcloned into lentiviral vectors enabling GFP and puromycin selection. Cas9- or dCas9-VPR–expressing B-ALL cells were cultured in RPMI-1640 with 20% FBS (Corning 35-010-CV), 55 μM β-mercaptoethanol (Gibco 21985023), and penicillin/streptomycin (Gibco 15140122), then infected with sgRNA libraries at low MOI (~0.3).

For Brunello and Metabolism-focused screens, each cell line was transduced with lentivirus encoding the sgRNA library and co-expression of GFP and a puromycin-resistance cassette (>500-fold sgRNA transduction coverage at multiplicity of infection [MOI] <1). For Brunello screen, after puromycin selection of GFP^+^ sgRNA^+^ cells for 14 days (allowing depletion of sgRNAs targeting essential genes), GFP^+^ cells were subjected to mock T or CD19 CAR-T cell co-culture for 72 h, at an Effector (T cell)-to-Target (B-ALL) (E:T) ratio optimized to elicit ~50% killing of each target cell line at this time point. For CRISPRa screens, cells were selected with puromycin (1 μg/mL, 72 h) from day 2, then split into lipid-replete or lipid-deplete media and challenged with IC50 CAR-T cells for 72 h. In all screens, surviving cells were harvested for sgRNA PCR. Following co-culture, we isolated genomic DNA to PCR amplify sgRNAs for deep sequencing. We calculated the average sgRNAs/gene, normalized counts to control sgRNAs, and compared sgRNA representation in Mock- versus CAR-T-treated conditions. Genes contributing to B-ALL resistance to CAR-T killing were defined as those for which corresponding sgRNAs exhibited significant depletion (log₂ fold-change <–0.5) in CAR-T–treated B-ALL cells relative to mock T-cell controls. Conversely, genes whose sgRNAs were enriched (log₂ fold-change > 0.5) under CAR-T treatment were considered to enhance sensitivity to CAR-T killing.

sgRNA libraries were amplified with ExTaq (Takara Bio) using: 95 °C 1 min; 26 cycles of 95 °C 30 s, 53 °C 30 s, 72 °C 30 s; and 72 °C 10 min. Custom staggered P5 and barcoded P7 primers (Supplementary Table 9) generated sequencing-ready libraries, which were run single-end 50 bp on HiSeq 4000. sgRNA counts were extracted from FASTQ files by identifying the 5’ CACCG motif and mapping the following 20 nt to the reference sgRNA list. Counts were normalized to CPM, log2-transformed (CPM + 1), and further normalized to non-targeting controls. Gene-level CAR-T–induced effects were calculated as the mean log2 fold-change (CAR/Mock) across all sgRNAs per gene.

### Quantitative real-time PCR analysis

Total mRNA was harvested from the indicated cell populations, using Qiagen RNeasy according to the manufacturer’s recommendations (Qiagen, #74004), followed by cDNA synthesis using the High-Capacity cDNA Reverse Transcription kit (ThermoFisher Scientific, #4368814). *SLC27A2* and *18S rRNA* expression was quantified by qPCR on QuantStudio 3 Real-Time PCR System (Applied Biosystems) by measuring SYBR Green (ThermoFisher Scientific, #A25742) incorporation. *SLC27A2* primers based on previously published primer design [51], with the following primer sequences: *SLC27A2* Forward 5’ TACTCTTGCCTTGCGGACTAA 3’, *SLC27A2* Reverse 5’ CCGAAGCAGTTCACCGATATAC 3’, *18S rRNA* Forward 5’ GCCGCTAGAGGTGAAATTCTTG 3’, *18S rRNA* Reverse ‘CTTTCGCTCTGGTCCGTCTT’.

### Immunoblotting

For immunoblot analysis, cell pellets were lysed using RIPA lysis and extraction buffer (Thermo Fisher, # 89900). The lysates were boiled with Laemmli buffer, resolved by SDS-PAGE, transferred to PVDF membranes and proteins visualized by immunoblotting. Antibodies used for immunoblotting analysis: p53 (R&D, Polyclonal, Cat# AF1355, RRID:AB_354749), p21 (Cell Signaling Technologies, Clone 12D1, Cat# 2947, RRID:AB_823586), FATP2 (Proteintech, Polyclonal, Cat# 14048-1-AP, RRID:AB_2239416) and beta-Actin (Cell Signaling Technologies, Clone 13E5, Cat# 4970, RRID:AB_2223172).

### Oil Red O staining

For histological analysis, 500,000 leukemic cells were isolated and resuspended in 80 µL PBS with 2% FBS. Cells were cytospun onto coated DoubleCytoslides (Shandon Cytospin 4; 400 rpm, 7 min) and air-dried for 2 h. Slides were immersed in Propylene Glycol (Abcam, ab150678) for 5 min, then incubated in Oil Red O Solution (Abcam, ab150678) overnight at room temperature, protected from air. The next day, slides were transferred to 85% Propylene Glycol for 1–2 min, rinsed twice in distilled water, stained in Hematoxylin (Abcam, ab150678) for 1–2 min, and rinsed thoroughly. After drying, coverslips (Fisher Scientific, 50-121-5160) were applied for microscopy.

### Electron microscopy

Cells were fixed overnight in 2.5% glutaraldehyde in 0.1 M sodium cacodylate buffer and then embedded in 3% agarose. Samples were trimmed into small blocks and rinsed three times in 0.1 M sodium cacodylate buffer. Post-fixation was performed with 2% osmium tetroxide and 0.8% K₃[Fe(CN)₆] in 0.1 M sodium cacodylate buffer for 1.5 h at room temperature. Cells were then rinsed with double-distilled water and en bloc stained with 4% uranyl acetate in 50% ethanol for 2 h. Following staining, samples were dehydrated in a graded ethanol series, infiltrated with Embed812 resin, and polymerized overnight at 70 °C. Blocks were sectioned using a diamond knife (Diatome) on a Leica UC7 ultramicrotome, collected onto copper grids, and post-stained with 2% aqueous uranyl acetate and lead citrate. Imaging was performed on a Tecnai T12 transmission electron microscope (Thermo Fisher) equipped with a LaB₆ source at 120 kV, with images acquired using an NS15 (15 Mpix) camera (AMT).

### Metabolomic and lipidomic profiling

For metabolomics, metabolites from frozen cell pellets were extracted in ice-cold 5:3:2 methanol:acetonitrile:water (v/v/v) at 2 × 10⁶ cells/mL by vigorous vortexing for 30 min at 4 °C, as described [52]. Global lipidomics of frozen pellets and supernatants used the same extraction conditions [53]. Extracts were clarified by centrifugation (10 min, 12,000 × *g*, 4 °C), randomized, and analyzed on a Thermo Vanquish UHPLC coupled to a Thermo Q Exactive or Orbitrap Exploris 120 MS. Metabolites and lipids were separated using 5-min C18 gradients [52, 54] and analyzed by electrospray ionization in positive and negative ion modes (two runs/sample) with QC every 10 injections. Lipidomics features were processed and integrated using LipidSearch (Thermo).

For isotope tracing, lipid-deplete media was supplemented with 5 µM each of ^13^C-Palmitic Acid (1,2,3,4-^13^C₄; CLM-7896), U-^13^C₁₈ Oleic Acid (CLM-460), and U-^13^C₁₈ Linoleic Acid (CLM-3960). B-ALL blasts were cultured for 72 h, after which supernatant and 100,000-cell pellets were collected, flash-frozen, and submitted for analysis. Remaining labeled cells were washed twice in lipid-replete media and subjected to Mock or CAR-T challenge (1:16 E:T) for 72 h, followed by sorting of 100,000 GFP⁺ cells, flash-frozen for analysis. Cell pellets were extracted in 100 µL cold 5:3:2 MeOH:ACN:H₂O; media aliquots were extracted 1:25 in the same solvent. Samples were vortexed 30 min at 4 °C and centrifuged (10 min, 18,213 rcf, 4 °C). Using 10 µL injections, metabolites were resolved using a high-throughput 5-min gradient [55], converted to. mzXML with RawConverter, and assigned/integrated using Maven with KEGG and an in-house standard library. Following metabolite extraction, 30 µL ice-cold MeOH was added to remaining pellets; media aliquots were extracted 1:25 in ice-cold MeOH and processed as above. For pellets, 30 µL metabolomics extract was combined with 30 µL MeOH prior to injection. Using 10 µL injections, non-polar lipids were analyzed by UHPLC-ddMS² using a 5-min method [53]. Lipidomics data were processed in LipidSearch 5.0 for lipid identification based on accurate mass, isotopic patterns, and fragmentation profiles. QC was assessed using technical replicates run at the beginning, middle, and end of each sequence [56].

### Imaging flow cytometry

B-ALL cells were co-cultured with either Mock or CAR-T cells at a 1:2 effector-to-target (E:T) ratio in lipid-replete media supplemented with 40 U/mL human IL-2 for 6 h. Cells were then fixed with 1% paraformaldehyde for 10 min on ice and washed three times with PBS containing 2% FBS. Surface and cytoskeletal staining was performed overnight at 4 °C using Human TruStain FcX (BioLegend, Cat# 422301, RRID:AB_2818986), AlexaFluor 647–conjugated Phalloidin (ThermoFisher Scientific, Cat# A22287), and PE-conjugated CD3 (Clone HIT3a, BioLegend, Cat# 300308, RRID:AB_314044) for Mock samples, or PE-conjugated anti-Whitlow/218 Linker (Clone E3U7Q, Cell Signaling Technology, Cat# 62405, RRID:AB_3626306) for CAR-T samples.

Following surface staining, cells were permeabilized using the FoxP3 Fix/Perm Buffer Set (BioLegend, Cat# 421403) and incubated for 2 h on ice with AlexaFluor 488–conjugated anti-GFP polyclonal antibody (Invitrogen, Cat# A-21311, RRID:AB_221477) and DAPI (Invitrogen, Cat# D1306). Cells were then washed twice with PBS + 2% FBS and acquired on the Amnis ImageStream®X Mk II using the 40× objective. Analysis was performed using IDEAS 6.4 software. Focused, single-cell events were gated based on area versus aspect ratio. Double-positive cells (GFP + CD3-PE+) were gated, and DAPI nuclear counts confirmed two-cell interactions. To exclude double GFP+ or double CD3+ cells, additional gating was applied on aspect ratio and area within the GFP channel. A synapse mask was generated by combining object and valley masks between DAPI signals. Synapse formation was quantified as the ratio of Phalloidin intensity per area within the synapse mask compared to the surrounding threshold mask. Synapse size and CD3 intensity per area within the synapse were measured. All samples were analyzed in batch using a standardized analysis template.

### Atomic force microscopy

B-ALL blasts cultured in lipid-replete media were subjected to AFM whereby JPK AFM and Bruker MSNL-10 tips were utilized to measure the Young’s Modulus of cells. C cantilever was calibrated in 37 °C serum media and verified to ~0.01 N/m under NanoWizard 3a Control Software (v6) with setpoint at 2 V and z-length at 3 µm. B-ALL were plated on RetroNectin (Cat# T100A, Takara) coated TPP AFM dishes (Cat#93040) and stored in cell incubator for at least two hours before the experiment. Same day cells measurement took place at 37 °C with serum media, setpoint at 1 nN and z-length set to 6 µm; ten cells were captured per condition per run with each cell measured at least five times with calibrated tip. Finally, data was analyzed using JPM data processing software (v6.0.77), fitting each force-distance curve using Hertz-Sneddon model with triangular pyramidal tip geometry to determine Young’s Modulus for each curve.

### St. Jude dataset analysis

We downloaded 1988 samples from the St. Jude PAX5-driven B-progenitor ALL dataset [29] on GenomePaint. Of these, 1104 patient samples contained survival data and were used to analyze *SLC27A2* expression based on FPKM values, which normalize for sequencing depth and gene length. Samples were stratified into “above-median” and “below-median” *SLC27A2* expression groups. Raw HTSeq read counts from these 1104 samples were processed with DESeq2 to perform differential expression analysis comparing the two groups. Genes were then ranked for Gene Set Enrichment Analysis (GSEA) using Hallmark gene sets, with top pathways selected based on the highest normalized enrichment scores (NES).

### Single cell RNA-seq analysis

We analyzed eight samples (four Mock- and four CAR-T–treated, 48 h) and processed Hashtag Oligonucleotide (HTO) data using Cell Ranger Multi v7.1.0 for sample demultiplexing via Multiplexing Capture feature barcodes. Resulting HDF5 files were merged into a single Seurat object for downstream analysis. Cell type annotation was performed using the BMCite anchoring workflow, followed by PCA and UMAP. Three major clusters were identified: a small T-cell cluster and two B-ALL clusters. Manual refinement removed PAX5⁺ B cells from the T-cell cluster and CD3D⁺ T cells from the B-ALL clusters, after which UMAP was recomputed. Within the B-cell compartment, cells were subdivided into Cluster 1 and Cluster 2. Differential expression analysis was performed using FindMarkers, followed by Hallmark gene set GSEA. In addition, Seurat Module Scores were computed for GOBP_FERROPTOSIS (M46862), GOBP_EXTRINSIC_APOPTOTIC_SIGNALING_PATHWAY (M11317), and GOBP_INTRINSIC_APOPTOTIC_SIGNALING_PATHWAY (M13922).

## Supplementary information

Supplementary Figures

Raw Western Blots

Supplementary Table 1

Supplementary Table 2

Supplementary Table 3

Supplementary Table 4

Supplementary Table 5

Supplementary Table 6

Supplementary Table 7

Supplementary Table 8

Supplementary Table 9

Supplementary Table 10

## Data Availability

Further information and requests for resources and reagents (e.g., human cell lines and plasmids) should be directed to Lead Contact, Matthew Witkowski (matthew.witkowski@cuanschutz.edu). PDX samples—SJHYPO009_D (“PDX1”) and SJHYPO003074_D2 (“PDX2”)—were obtained through the St. Jude PROPEL resource under a Materials Transfer Agreement. This work was supported in whole or in part by NIH funding and is subject to the NIH Public Access Policy, which grants the NIH the right to make the work publicly available in PubMed Central. Sequencing data have been deposited in the NCBI Gene Expression Omnibus under accession GSE291834. Organized data are publicly available through the Open Science Framework: osf.io/ra8mv.

## Code availability

Code used for scRNA-sequencing analysis is publicly available at GitHub: https://github.com/Tzuinformatics.
