# Supplementary material for: FATP2-mediated lipid metabolism enhances chimeric antigen receptor T-cell therapy resistance in B-cell acute lymphoblastic leukemia
Source: Leukemia. 2026 Jun 30;40(8):1763–77. doi: 10.1038/s41375-026-03030-0 (PMC13421331; doi:10.1038/s41375-026-03030-0)
Supplement: Supplementary file 1 — Supplementary Figures [file 41375_2026_3030_MOESM1_ESM.pdf]

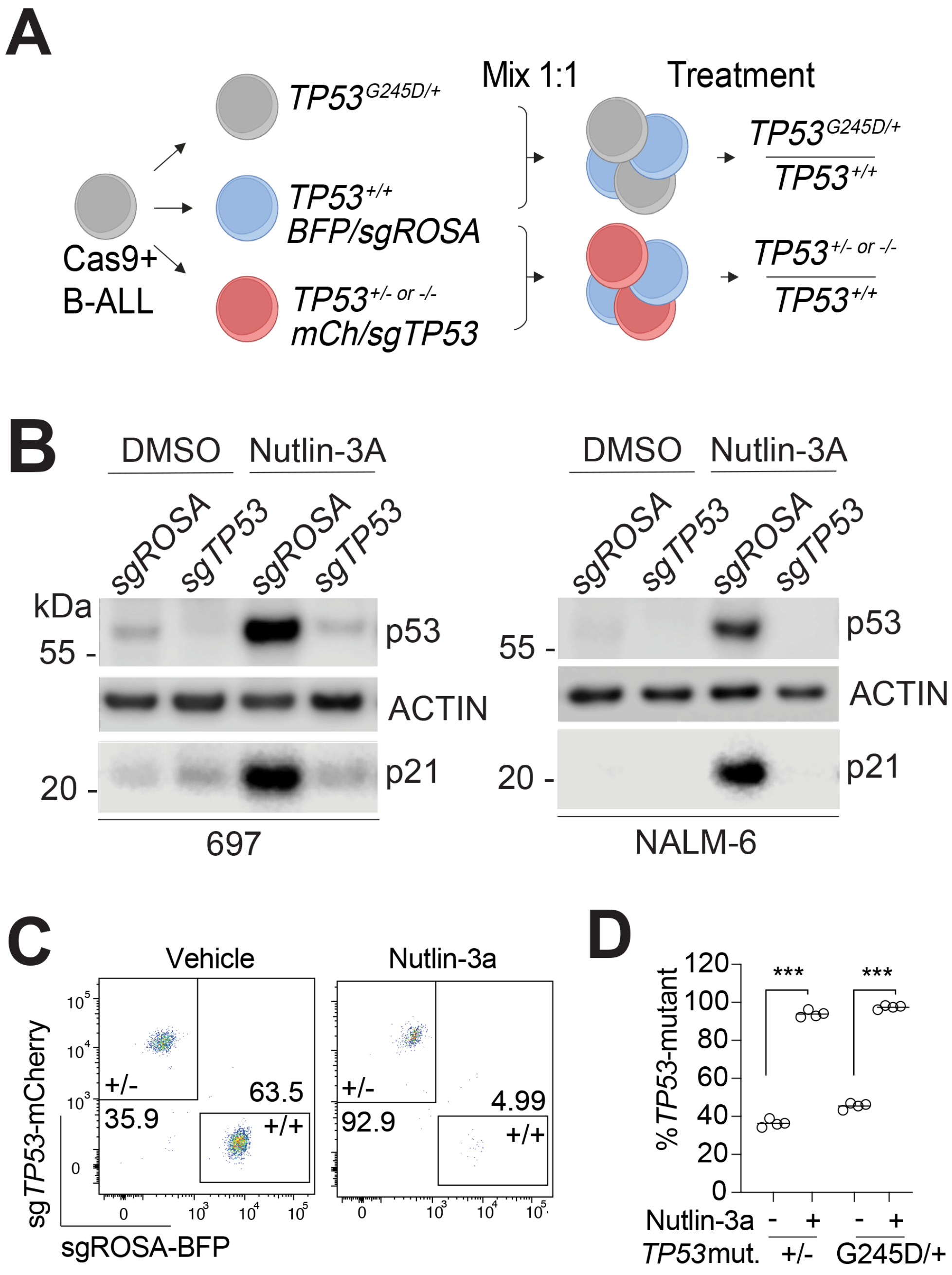

Supplementary Figure 1. (A) Schematic of *TP53*-targeted editing of Cas9-expressing B-ALL and *TP53*-mutant:*TP53*-wildtype competition assay. **(B)** Immunoblot of p53, p21, and β-Actin for sg*ROSA*-BFP (*TP53*-wildtype) and *sgTP53*-mCherry (*TP53*+/-, *TP53*-mutant) 697 and NALM-6 cells treated with Nutlin-3A (10 μM) for 4 hours. **(C)** Flow cytometry of 697 *TP53*-wildtype (+/+) and *TP53*-mutant (+/-) competition following 72 hours vehicle (DMSO) or Nutlin-3a (10 μM) treatment. **(D)** Histogram of %*TP53*-mutant cells upon Nutlin-3a treatment (vehicle normalized) for 697 *TP53*-mutant:*TP53*-wildtype competition. *TP53* genotype shown. 2-way ANOVA, n = 4 replicates, mean shown.

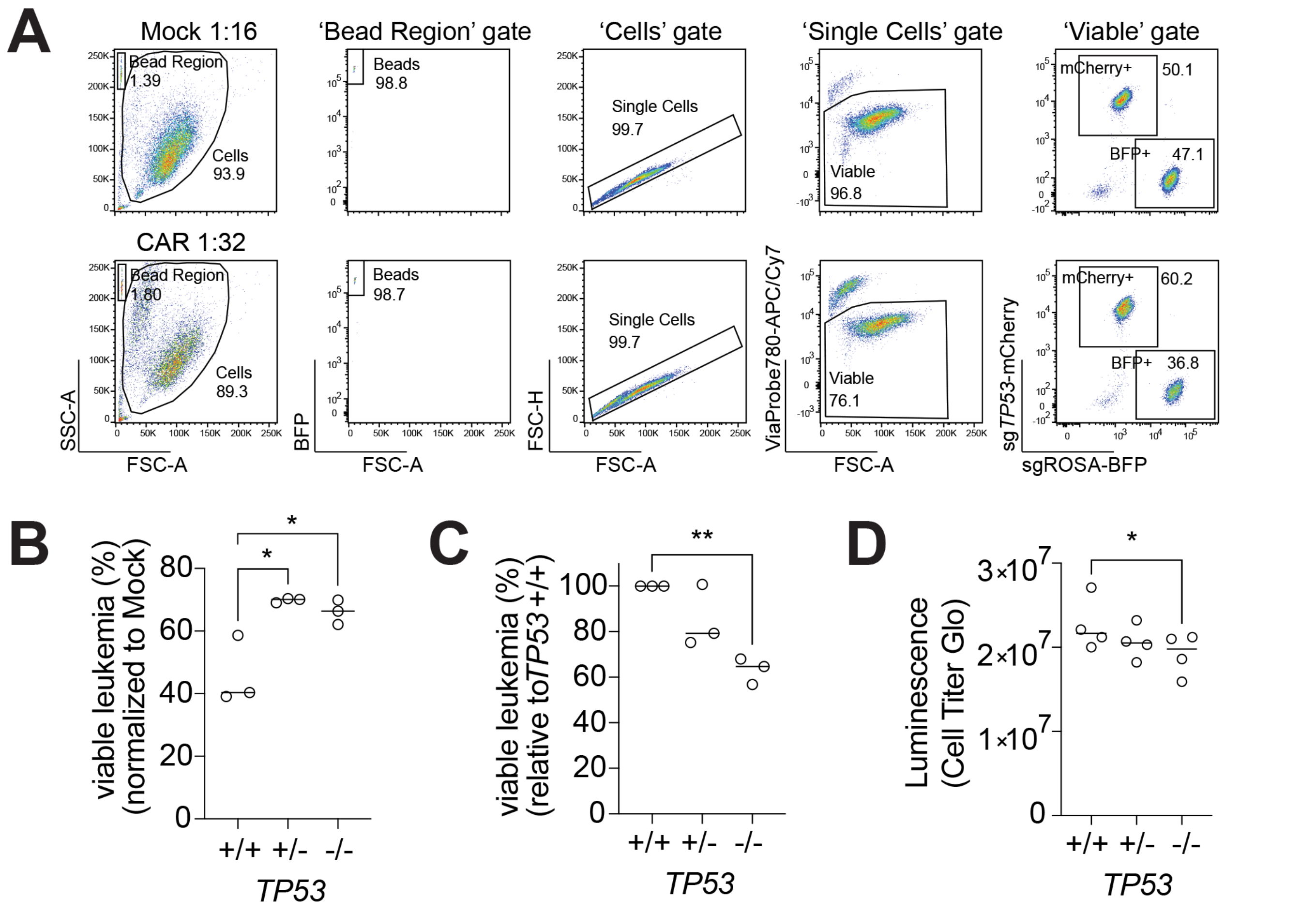

Supplementary Figure 2. (A) Representative flow cytometry gating strategy for 697 *TP53*-mutant:*TP53*-wildtype *in vitro* competition assays. The analysis includes sequential gating of counting beads, mCherry+ and BFP+ B-ALL subpopulations, and assessment of surface CD19 expression under Mock or CAR-T treatment conditions. Effector-to-target (E:T) ratios are indicated. **(B)** Live leukemia count (normalized to Mock control) following Mock or CD19 CAR-T cell treatment (1:16 E:T) of *TP53*-wildtype (+/+) and *TP53*-mutant (+/-, -/-) 697 B-ALL following 72 hours. 2-way ANOVA versus. n = 3 independent experiments, individual values and mean shown. **(C-D) (C)** Live leukemia count and **(D)** metabolic activity (Cell Titer Glo luminescence) of *TP53*-wildtype (+/+) and *TP53*-mutant (+/-, -/-) 697 B-ALL following 72 hours cell culture following seeding of equal cell numbers. 2-way ANOVA versus. n = 3 independent experiments, individual values and mean shown.

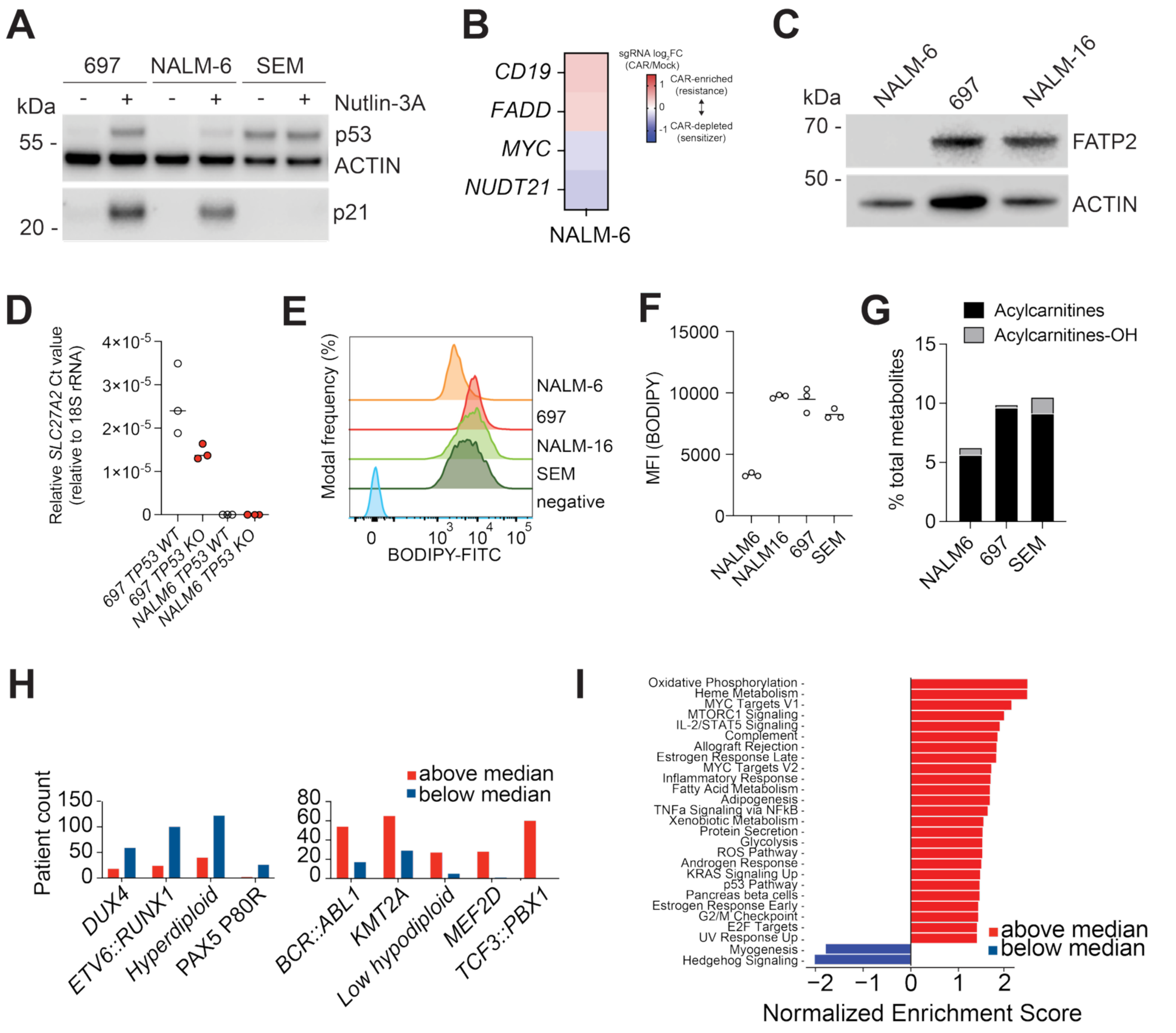

Supplementary Figure 3. (A) Immunoblot of p53, p21, and β-Actin for 697, NALM-6 and SEM cells treated with Nutlin-3a (10uM) for 4 hours. **(B)** Heatmap showing $\log_2$fold-change of gene-specific sgRNAs comparing Brunello library-transduced Cas9$^+$ NALM-6 cells challenged with CAR-T *versus* Mock T sgRNA representation normalized to non-targeting control sgRNAs. **(C)** Immunoblot of FATP2 and β-Actin for NALM-6, 697 and NALM-16 B-ALL. **(D)** Quantitative real-time PCR analysis of *SLC27A2* mRNA expression (relative to 18S rRNA housekeeper control) for *TP53*-wildtype and *TP53*-mutant 697 and NALM-6 B-ALL. Ct values, n = 3 independent experiments, individual points shown. **(E)** Representative flow cytometry histogram of BODIPY uptake comparing NALM-6, 697, NALM-16 and SEM B-ALL lines. **(F)** Quantification of BODIPY uptake across B-ALL cell lines. ^{13}C-BODIPY mean fluorescence intensity (MFI). n = 3 independent experiments. **(G)** Histogram showing percentage acylcarnitines of total metabolites for NALM-6, 697 and SEM B-ALL based on global metabolomics analysis. Median of two independent replicates shown. **(H)** Histogram of B-ALL subtype-specific patient numbers in *SLC27A2*^{low} and *SLC27A2*^{high} groups (St. Jude dataset). **(I)** Gene set enrichment analysis of differentially expressed genes when comparing *SLC27A2*^{low} and *SLC27A2*^{high} groups (St. Jude dataset). Top 25 enriched pathways shown.

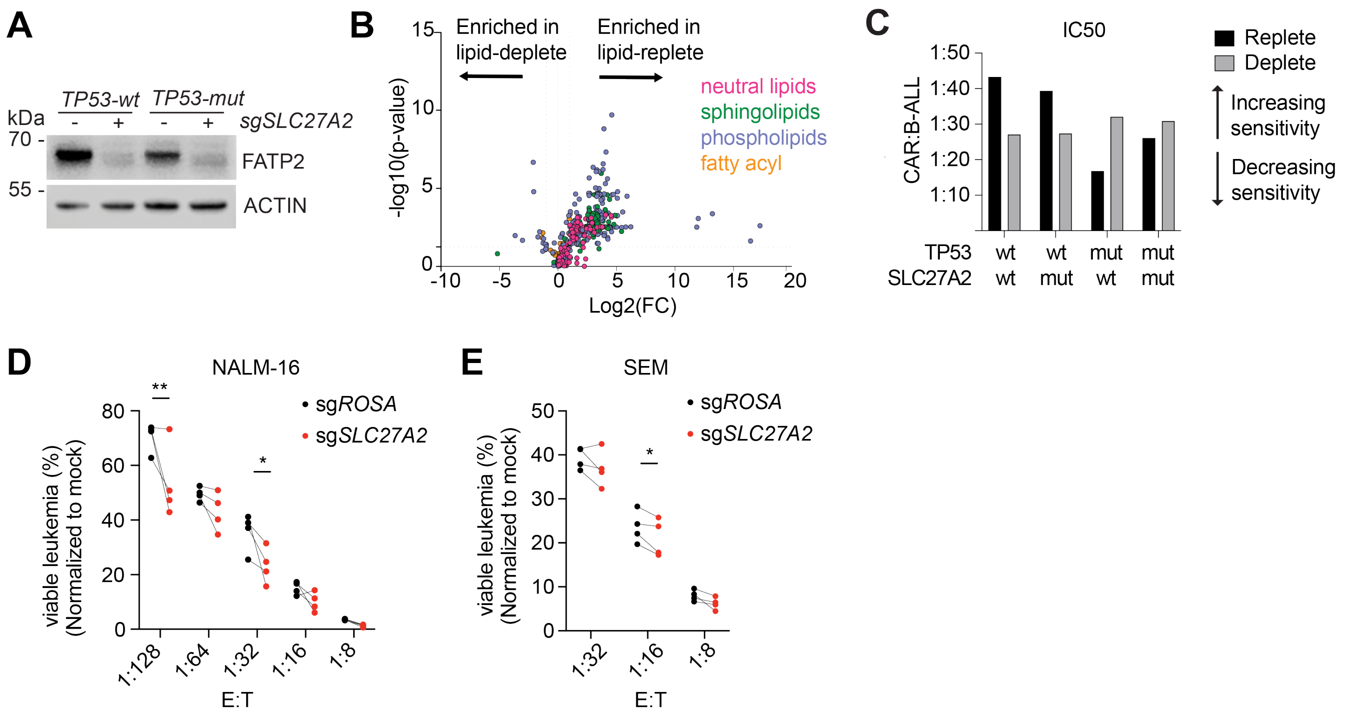

Supplementary Figure 4. (A) Immunoblot analysis of FATP2 and β-Actin for *TP53*-wildtype and *TP53*-mutant 697 B-ALL ± sg*SLC27A2* or *sgROSA* expression. **(B)** Volcano plot of global lipidomic analysis comparing lipid-replete and lipid-deplete media. Lipid classes are color coded on plot. n = 6 replicates per condition, axis displaying $\log_2$fold-change and $\log_{10}$(p-value). **(C)** Histogram of CAR-T IC_{50} values for isogenic 697 B-ALL challenged with CD19 CAR-T cells under lipid-replete or lipid-deplete conditions with Effector:Target ratio shown. **(D-E)** CD19 CAR-T cell treatment of **(D)** NALM-16 and **(E)** SEM B-ALL-expressing sg*ROSA* or sg*SLC27A2* for 72 hours. 2-way ANOVA versus sg*ROSA*, * $p < 0.05$, ** $p < 0.01$, n=4 replicates at indicated CAR:target ratios. % viable leukemia normalized to Mock T control is shown.

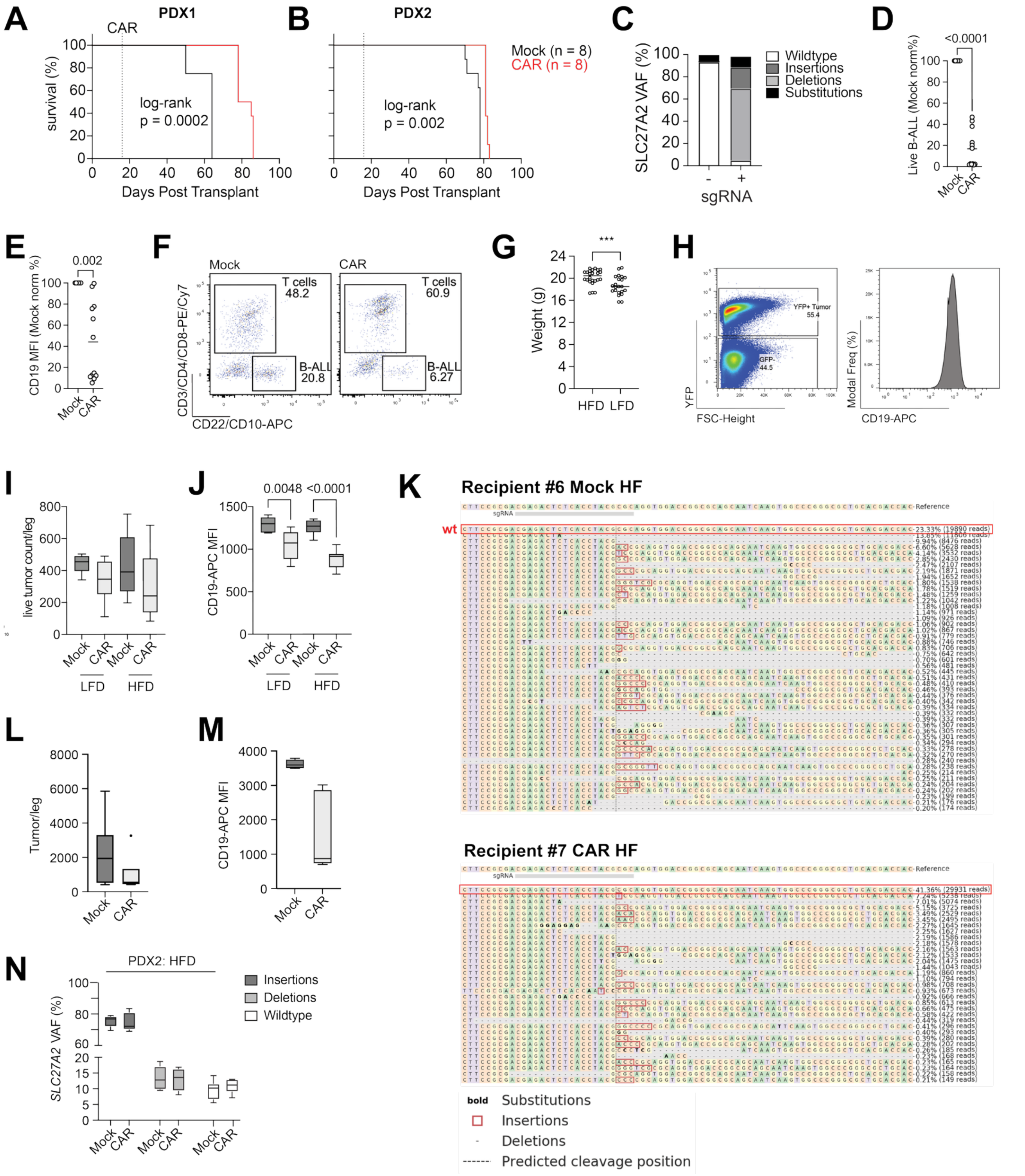

Supplementary Figure 5. (A-B) Kaplan-Meier curves of **(A)** PDX1 and **(B)** PDX2 overall survival following infusion of 100,000 Mock or CAR-T cells at 14 days post-engraftment. Log-rank test, p value shown. n = 8 recipients (4 male, 4 female). **(C)** Histogram showing *SLC27A2* Exon 1-specific gene editing events in 697 B-ALL cells as measured by amplicon sequencing. **(D-F)** Quantification of **(D)** absolute DAPI^{-}YFP^{+} cell counts, **(E)** surface CD19 mean fluorescence intensity of YFP^{+} cells, and **(F)** Representative flow cytometry of PDX1 B-ALL (CD10, CD22 pooled antibody mix) and T cell (CD3, CD4, CD8 pooled antibody mix) (Mock or CAR) following 48-hours co-culture on MS-5 stroma. **(G)** Histogram of 4-6-week-old NSG mice weight (g) following 1-week of LFD and HFD. **(H)** Representative flow cytometry of Mock-treated PDX1 recipient bone marrow at time of harvest, showing YFP^{+} B-ALL tumor, and YFP^{+} CD19 expression. **(I-J)** Quantification of PDX1 **(I)** absolute DAPI^{-}YFP^{+} cell counts in recipient bone marrow, **(J)** surface CD19 mean fluorescence

intensity of YFP^+ cells. n = 5-6 recipients per group. Tukey box plot, 1-way ANOVA, p-values shown. **(K)** Representative raw amplicon sequencing data displaying targeted *SLC27A2* exon 1 region for Mock and CAR-T-treated recipients. Wildtype sequences matching the reference wildtype highlighted in red. **(L-M)** Quantification of PDX2 **(L)** absolute $DAPI^-YFP^+$ leukemia cell counts in recipient bone marrow, **(M)** surface CD19 mean fluorescence intensity of YFP^+ cells under HFD conditions. n = 6-9 recipients per group. Tukey box plot. **(N)** Frequency of *SLC27A2* variant alleles (measured by targeted amplicon sequencing of sorted YFP+ leukemia) reads bone marrow PDX2 B-ALL blasts subjected to Mock/CAR treatment under HFD.

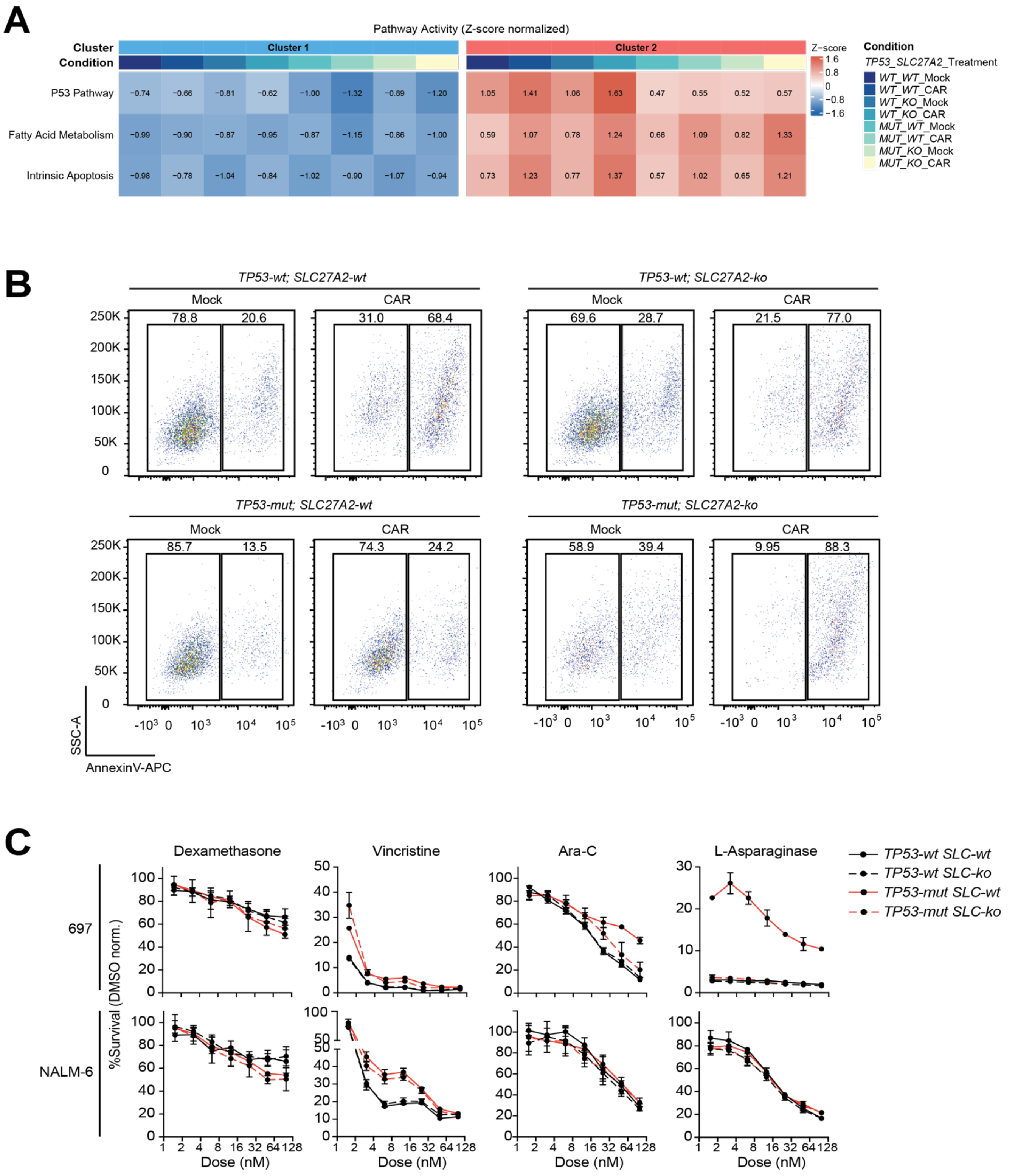

Supplementary Figure 6. (A) Seurat Module Scores (Pathways: p53 pathway, Fatty Acid Metabolism and Intrinsic Apoptosis) for Cluster 1 and Cluster 2 isogenic 697 B-ALL cell lines following Mock or CAR-T exposure. Z-score normalization of each pathway across all samples shown. **(B)** Representative flow cytometry of Annexin V staining of B-ALL lines (genotypes indicated) following 72 hours Mock or CAR-T exposure (E:T 1:16). **(C)** Cell viability following 72-hours treatment with Dexamethasone, vincristine, Ara-C and L-Asparaginase treatment of isogenic 697 and NALM-6 B-ALL, with *TP53* and *SLC27A2* genotype shown. Cell viability measured by Cell Titer Glo assay, n = 4 independent experiments at indicated drug doses. % viable leukemia normalized to DMSO control is shown.

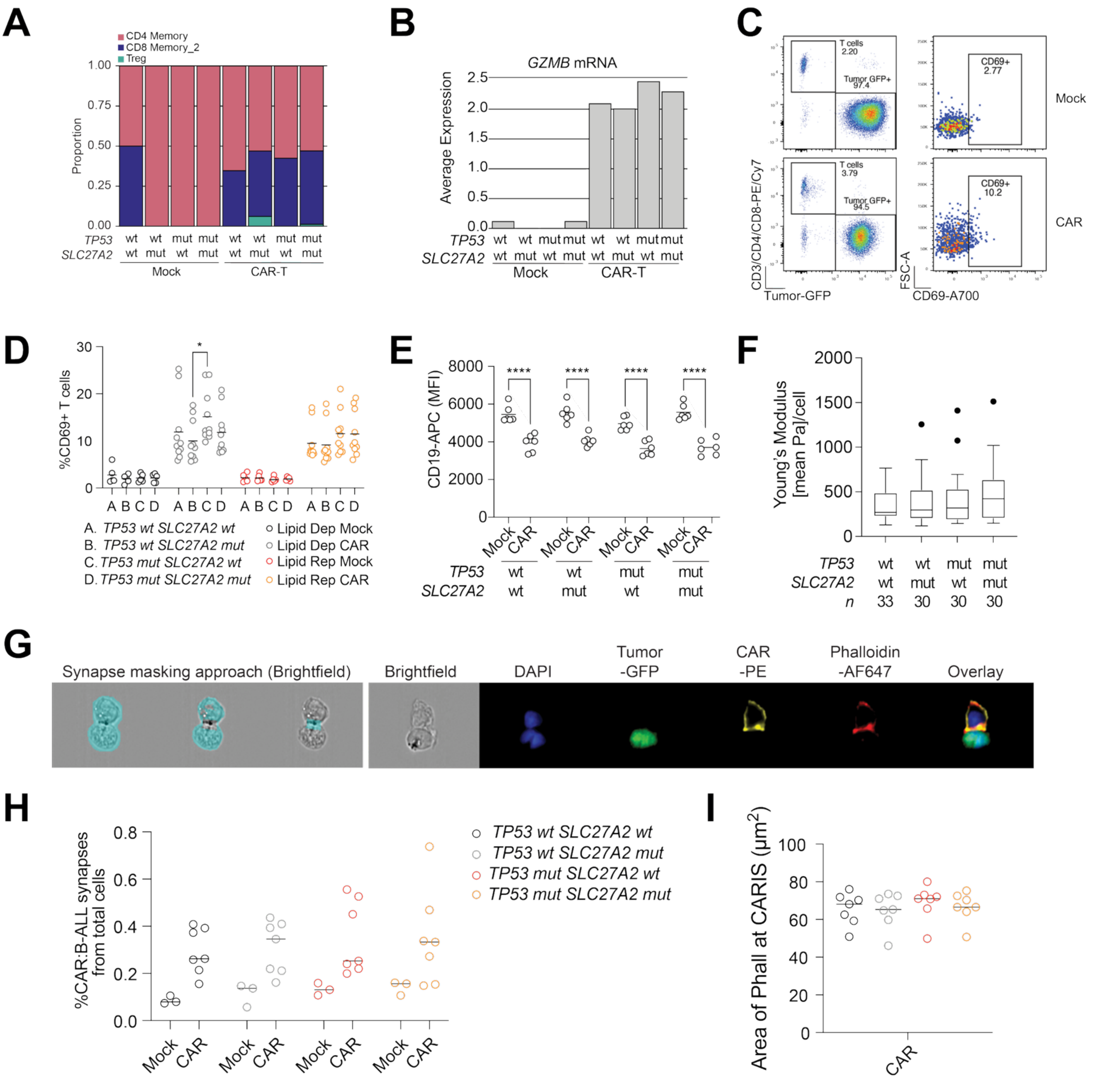

Supplementary Figure 7. (A) Histogram of the percentage of Seurat-defined T cell subsets, for each genotype and T cell treatment shown. **(B)** Histogram of *GZMB* mRNA expression in T cells co-cultured with isogenic B-ALL cell lines (genotypes indicated) under Mock or CAR-T treatment conditions. **(C-D) (C)** Representative flow cytometry and **(D)** histogram of %CD69^{+} T cells (CD3^{+}CD4^{+}/CD8^{+}; pooled antibody mix) co-cultured with isogenic B-ALL cell lines (genotypes indicated) for 72 hours at a 1:25 E:T ratio. 2-way ANOVA, with individual replicates shown, *p < 0.05. **(E)** Surface CD19 expression histogram of isogenic 697 B-ALL cells (genotypes shown) co-cultured with Mock or CAR T cells for 72 hours at a 1:25 E:T ratio. 2-way ANOVA, with individual replicates shown, ****p < 0.0001. **(F)** Young's Modulus Calculation of isogenic 697 B-ALL cell lines (genotype indicated) subjected to Atomic Force Microscopy. Number of individual cells analyzed (n) shown. Tukey box plot. **(G)** Representative imaging flow cytometry of GFP^{+} B-ALL interacting with single CAR^{+} T cells. Panels show masking strategy to identify synapse region, followed by representative single channels showing DAPI (DNA), GFP (Tumor), CAR-specific linker-PE, phalloidin-AlexaFluor647, and overlay. **(H)** Histogram showing percentage single GFP^{+} tumor:T cell (CAR or Mock) interactions relative to total events analyzed using imaging cytometry. n = 3-6 independent co-cultures. Cell line genotypes and individual data points with mean shown. **(I)** Histogram showing area of phalloidin-AlexaFluor647 staining (µm^{2}) within synapse mask at the CAR immunological synapse (CARIS). Cell line genotypes and individual data points with mean shown.

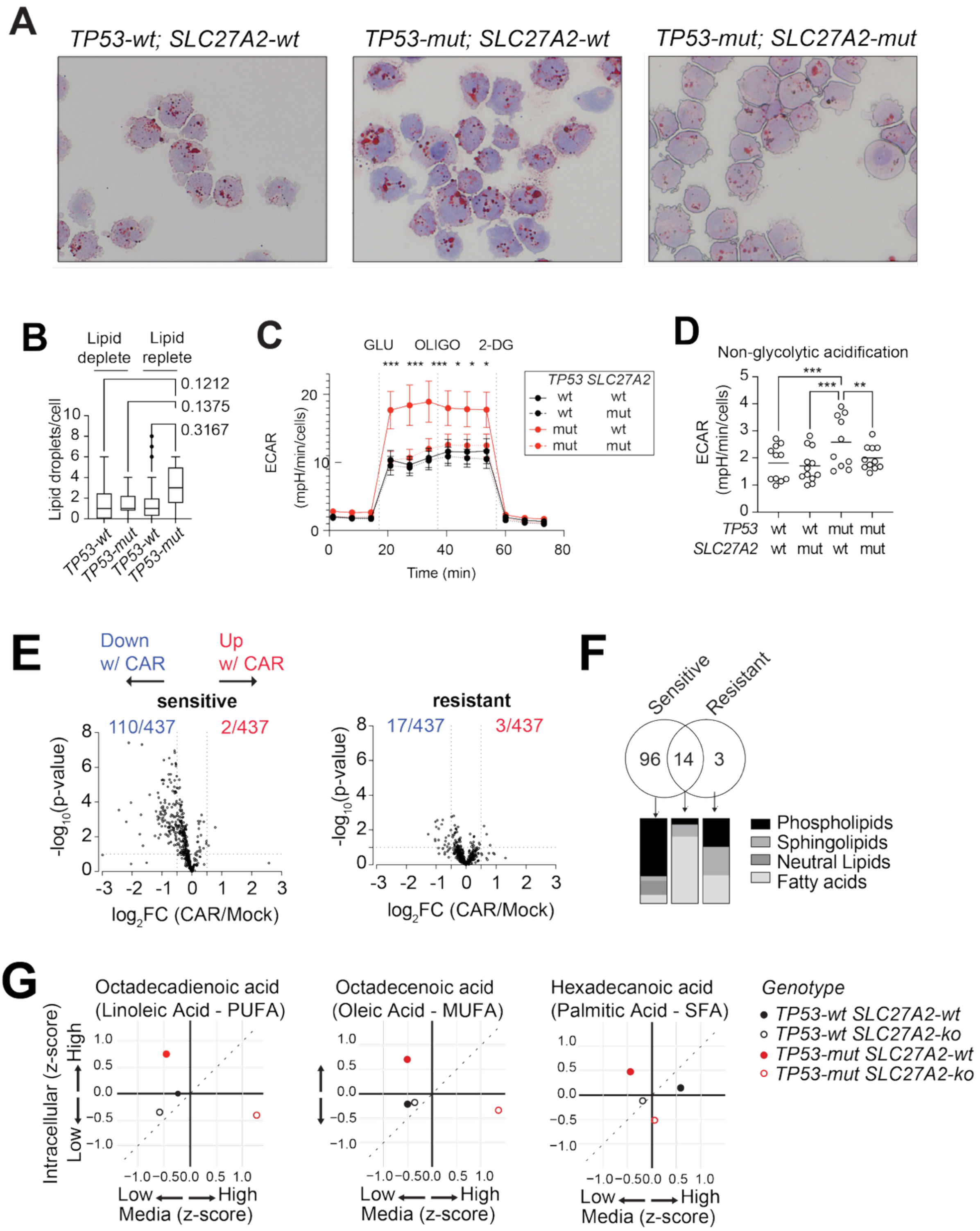

Supplementary Figure 8. (A) Representative Oil Red O staining images across isogenic 697 B-ALL lines. **(B)** Histogram quantification of lipid droplets per cells using electron microscopy for *TP53*-wildtype and *TP53*-mutant 697 B-ALL with lipid droplets indicated. Two independent experiments, n = individual single cells analyzed, 2-way ANOVA performed. **(C)** Metabolic flux assays to measure glycolytic rate (extracellular acidification rate; ECAR) for all isogenic 697 B-ALL cells. Statistical analysis was performed using two-way ANOVA to determine relative ECAR was determined for *TP53*-mutant; *SLC27A2*-wildtype relative to *TP53*-mutant; *SLC27A2-knockout* shown at each time point. **(D)** Non-glycolytic acidification measurement derived from metabolic flux assays to measure glycolytic rate (extracellular acidification rate; ECAR) for all isogenic 697 B-ALL cells. 2-way ANOVA to determine relative ECAR was determined for *TP53*-mutant; *SLC27A2*-wildtype relative to *TP53*-mutant; *SLC27A2*-mutant shown at each time point. **(E)** Volcano plot of differentially represented lipid species comparing CAR- versus Mock-treated isogenic 697 B-ALL lines grouped as

‘Resistant’ (*TP53*-mutant; *SLC27A2*-wildtype) and ‘Sensitive’ (*TP53*-wildtype; *SLC27A2*-wildtype, *TP53*-wildtype; *SLC27A2-knockout*, *TP53*-mutant; *SLC27A2-knockout*). Cut-offs: $\log_2$fold-change > ±0.5, $-\log_{10}$(p value > 1). **(F)** Venn diagram representation of the number of overlapping lipid species and histogram of lipid classes specific to downregulated lipids following CAR-T treatment of CAR-sensitive versus CAR-resistant, Mock treatment (n =3), CAR (n = 6: n = 3/T cell donor, two independent T cell donors). **(G)** Scatterplot showing the average z-score of intracellular (y-axis) and supernatant media (x-axis) ^{13}C-FA levels for labeled linoleic, oleic and palmitic acid following 72-hours of isogenic 697 B-ALL cell culture in ^{13}C-FA-containing media. Average based on four independent experiments.

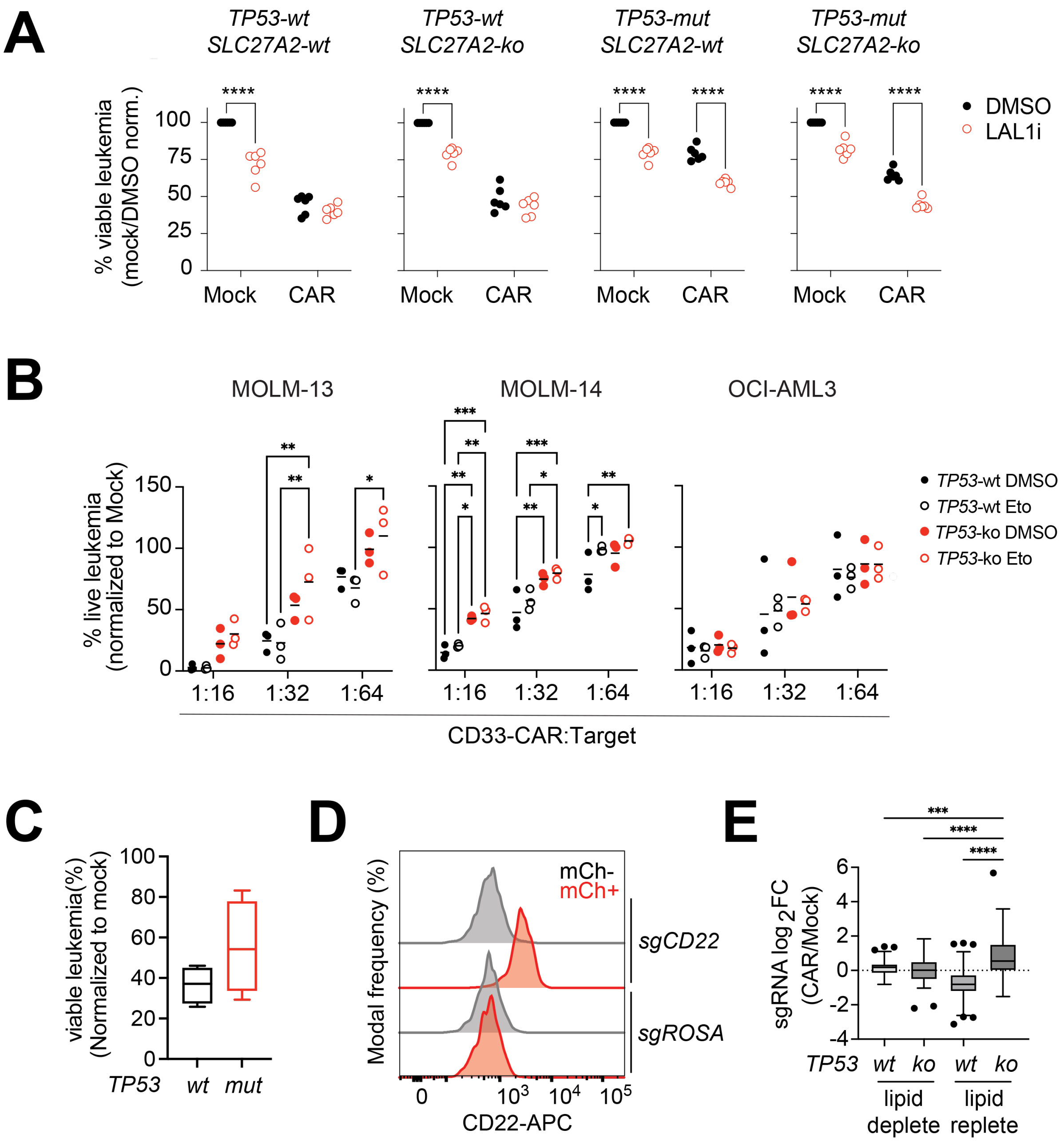

Supplementary Figure 9. (A) Percentage live leukemia (normalized to Mock/DMSO control) following Mock or CD19 CAR-T cell treatment (1:16 E:T) of *TP53*-wildtype and *TP53*-mutant 697 B-ALL for 72 hours with co-treatment of DMSO control and/or lalistat-1 (10 μM). 2-way ANOVA versus DMSO. n = 5 replicates. Individual points with mean shown. **(B)** Percentage viable leukemia (relative to Mock T cells controls) following CD33 CAR-T cell treatment of *TP53*-wildtype and *TP53*-mutant MOLM-13, MOLM-14 and OCI-AML3 AML for 72 hours in the presence or absence of etomoxir (5 μM). E:T ratios shown. 2-way ANOVA. n=3 independent experiments, Individual points with mean shown. *p < 0.05, **p < 0.01, ***p < 0.005.**(C)** Percentage live leukemia (normalized to Mock/DMSO control) following Mock or CD19 CAR-T cell treatment (1:16 E:T) of *TP53*-wildtype and *TP53*-mutant dCas9-VPR+ 697 B-ALL. n = 4 replicates. Tukey box/whisker plot shown. **(D)** Representative flow cytometry histograms of CD22 surface expression of *TP53*-wildtype and *TP53*-mutant dCas9-VPR+ 697 B-ALL expressing sgROSA or sgCD22. **(E)** Histogram quantification of individual fatty acid oxidation-specific sgRNA representation (Gene Ontology:0006635) comparing Human Metabolic Genes CRISPRa sgRNA library-transduced, isogenic dCas9-VPR 697 *TP53*-wildtype and *TP53*-mutant B-ALL cell lines challenged with CAR-T *versus* Mock T under lipid replete and deplete culture conditions, with sgRNA representation normalized to non-targeting control sgRNAs.
