# Supplementary material for: FATP2-mediated lipid metabolism enhances chimeric antigen receptor T-cell therapy resistance in B-cell acute lymphoblastic leukemia
Source: Leukemia. 2026 Jun 30;40(8):1763–77. doi: 10.1038/s41375-026-03030-0 (PMC13421331; doi:10.1038/s41375-026-03030-0)

Figure 2B Raw Blots

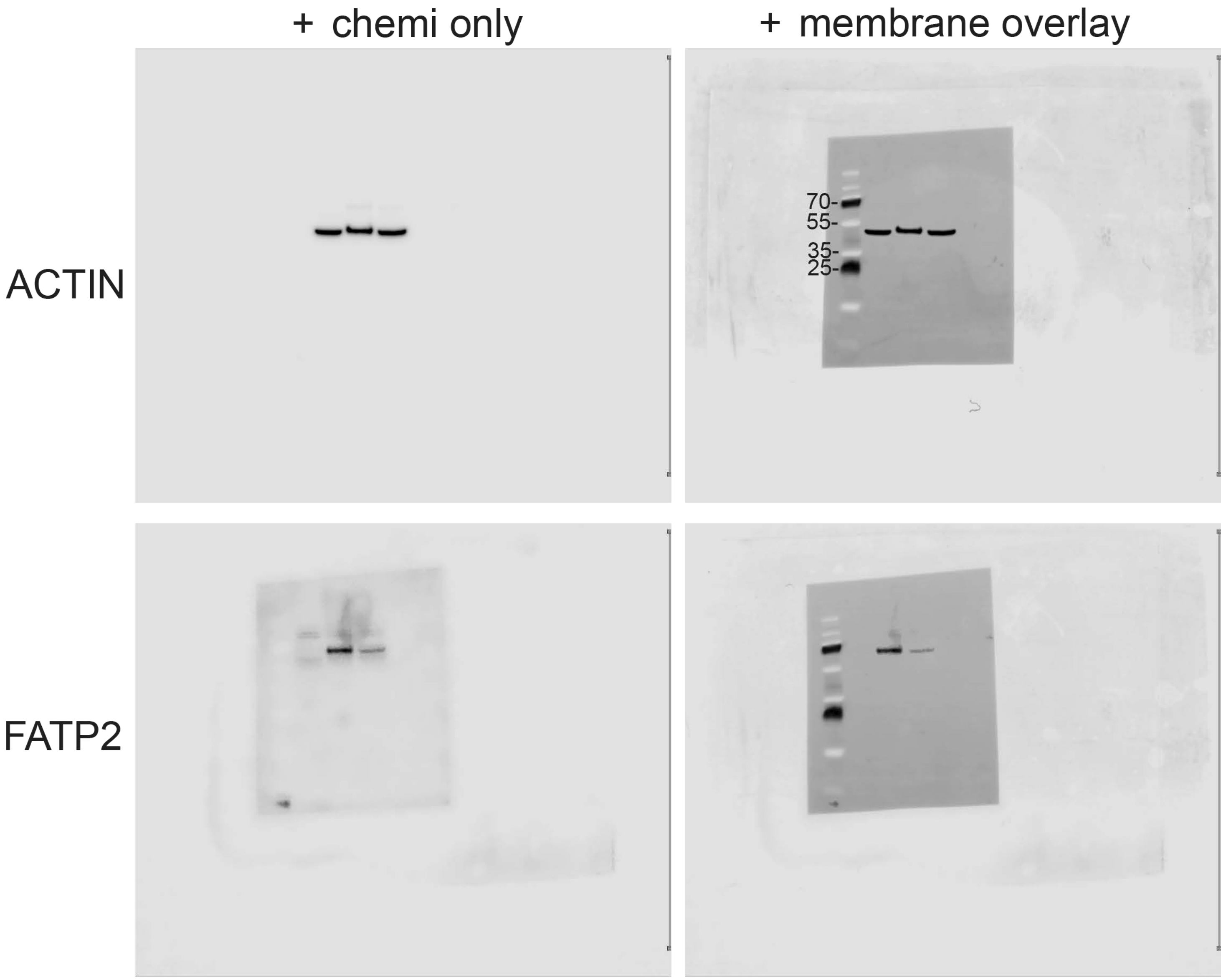

Final Figure

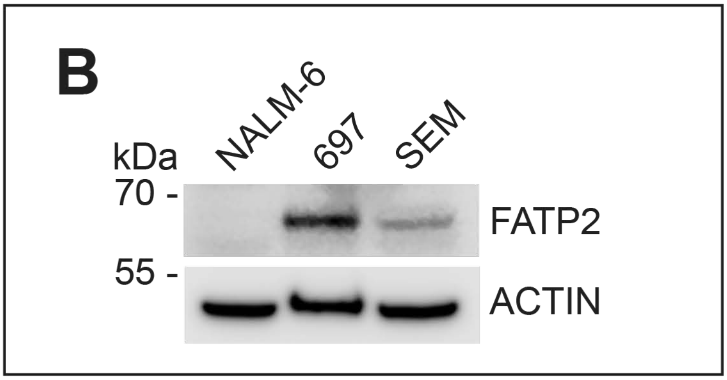

Figure 3C Raw Blots

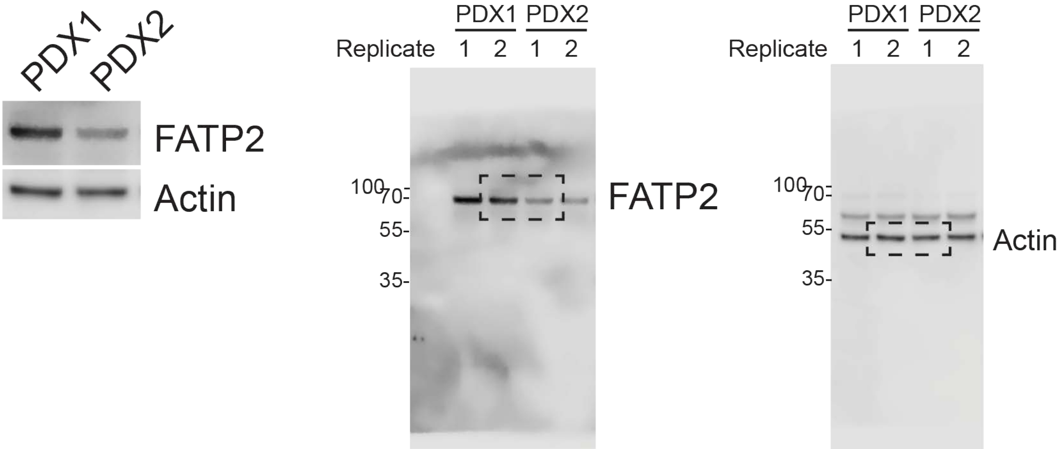

Figure 3E Raw Blots

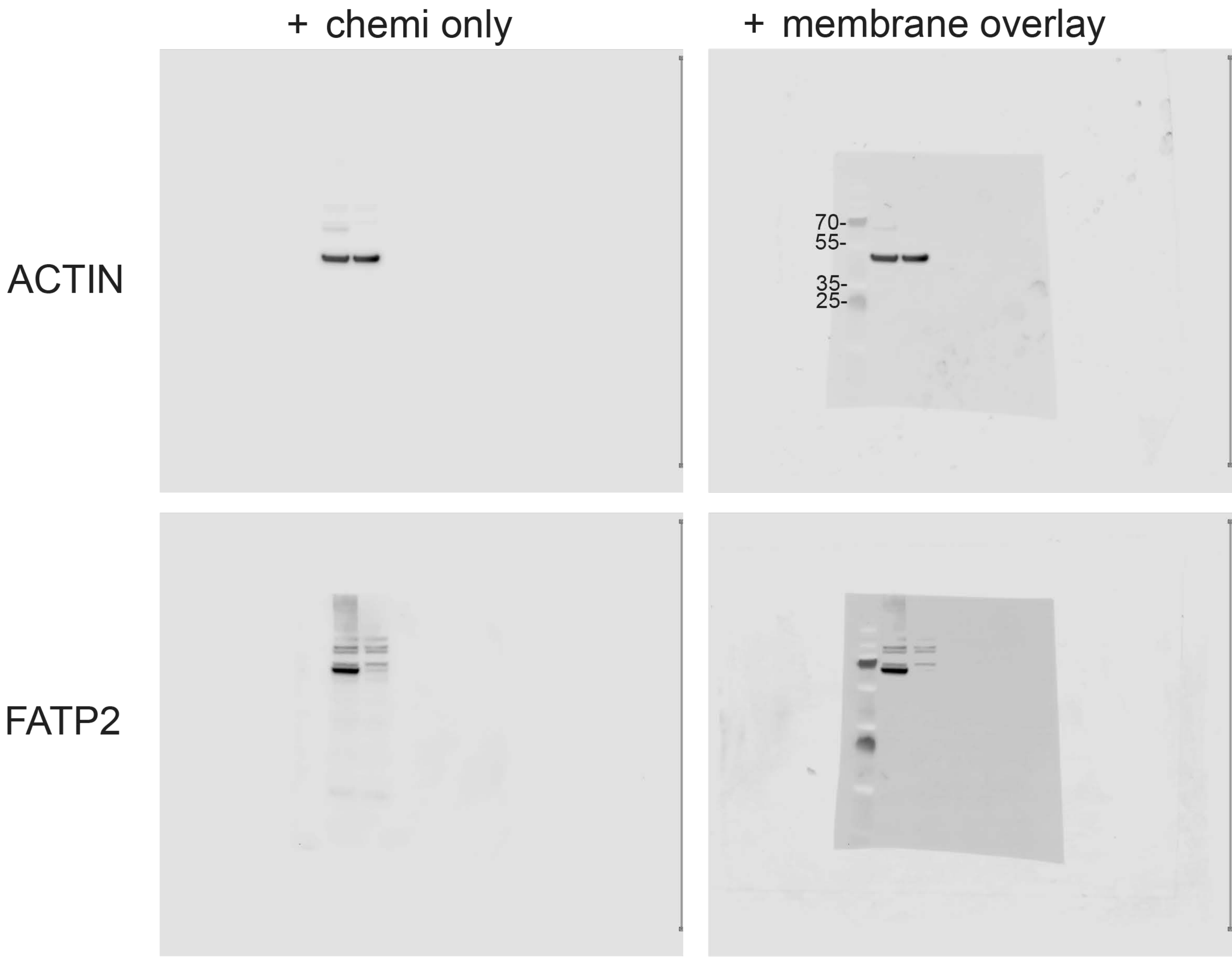

Final Figure

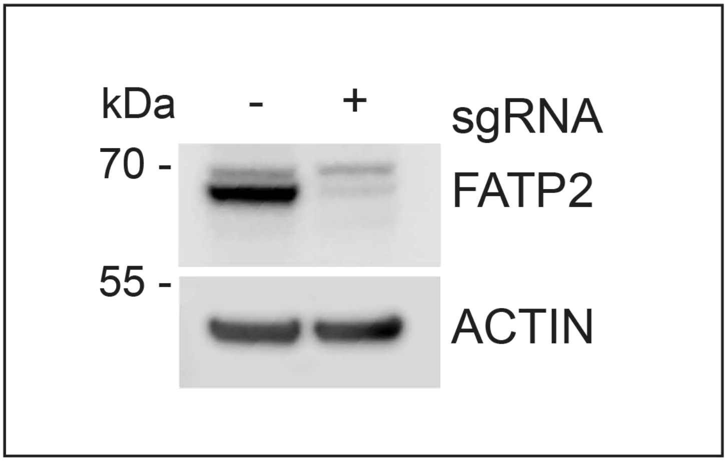

Supplementary Figure 1B Raw Blots

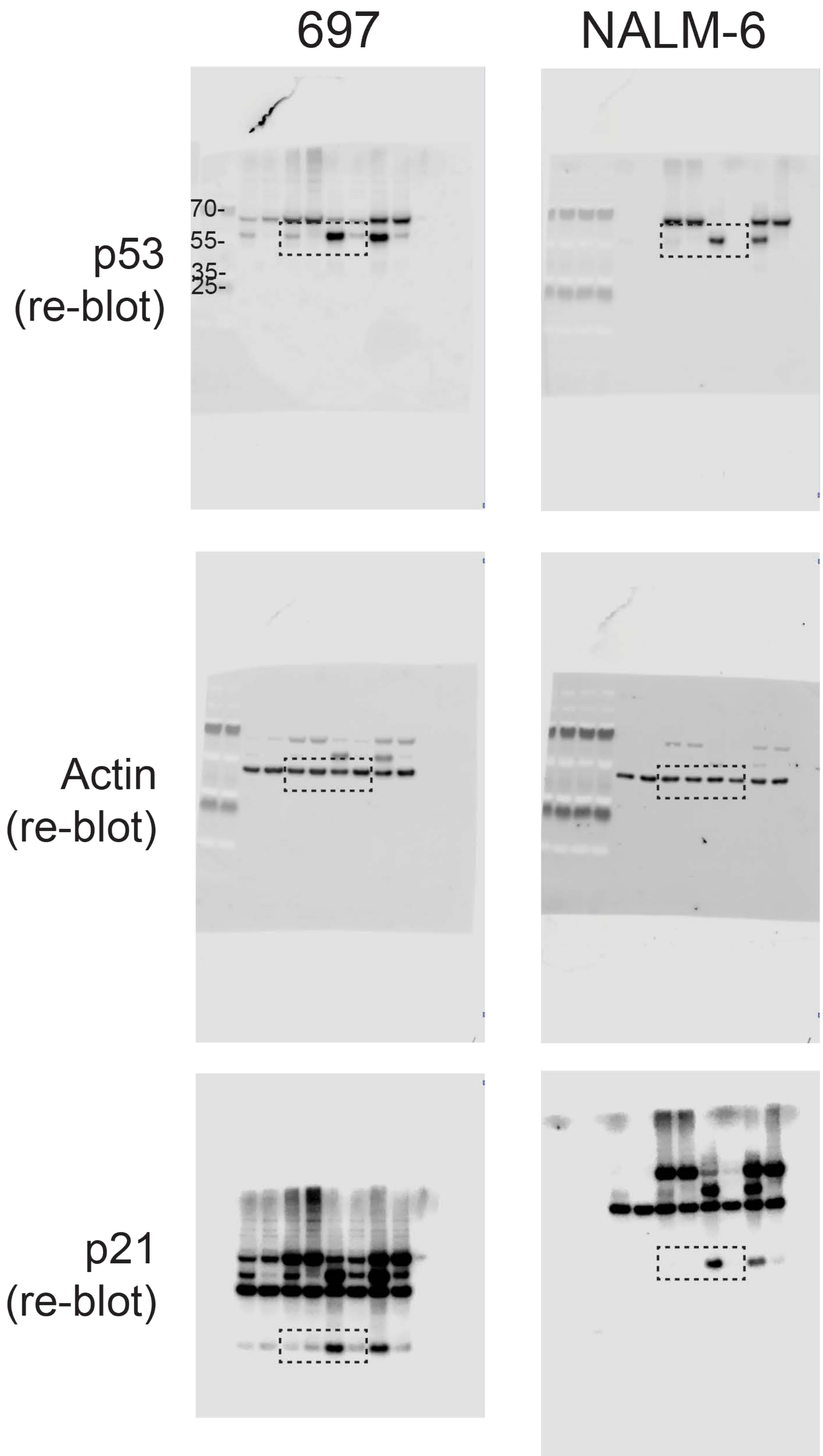

Final Figure

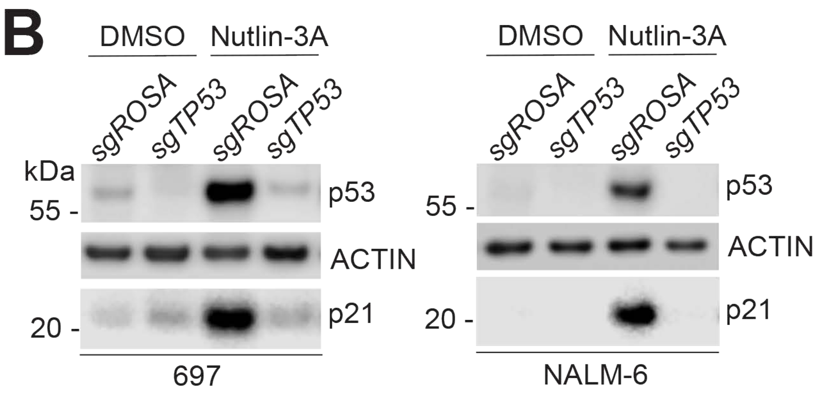

Supplementary Figure 3A Raw Blots

\+ chemi only

\+ membrane overlay

p53
ACTIN
p21
(long exp)

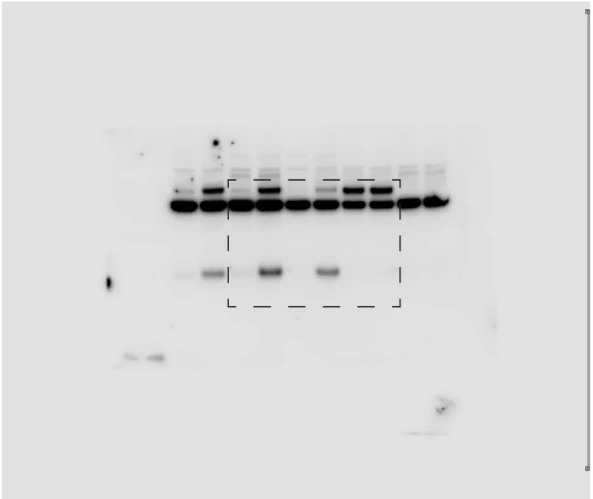

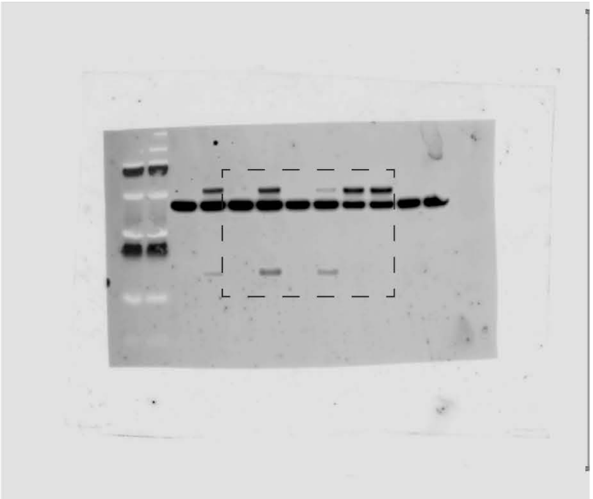

p53
ACTIN
p21
(short exp)

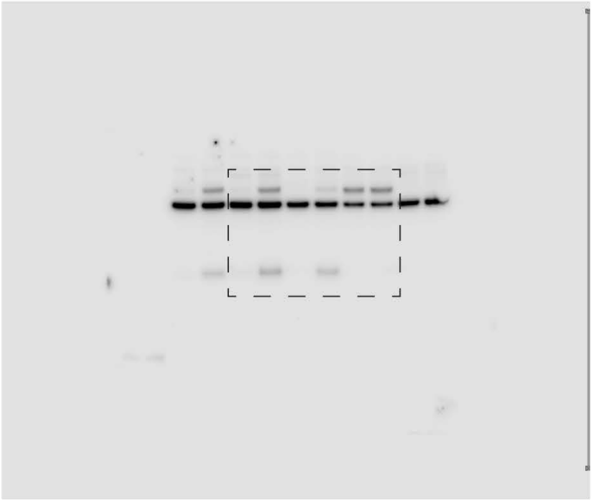

Final Figure

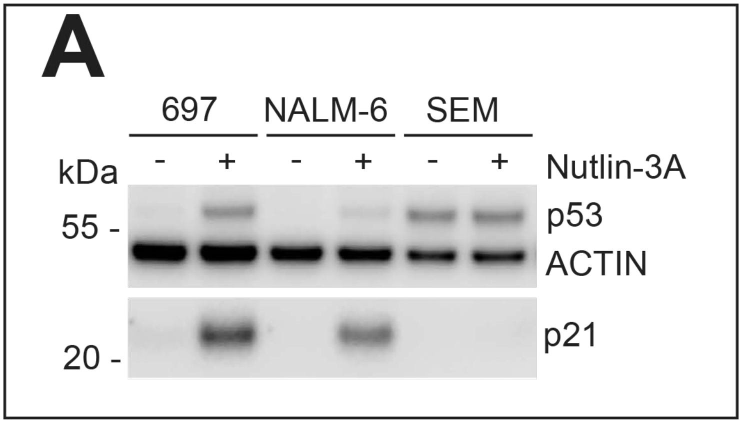

Supplementary Figure 3C Raw Blots

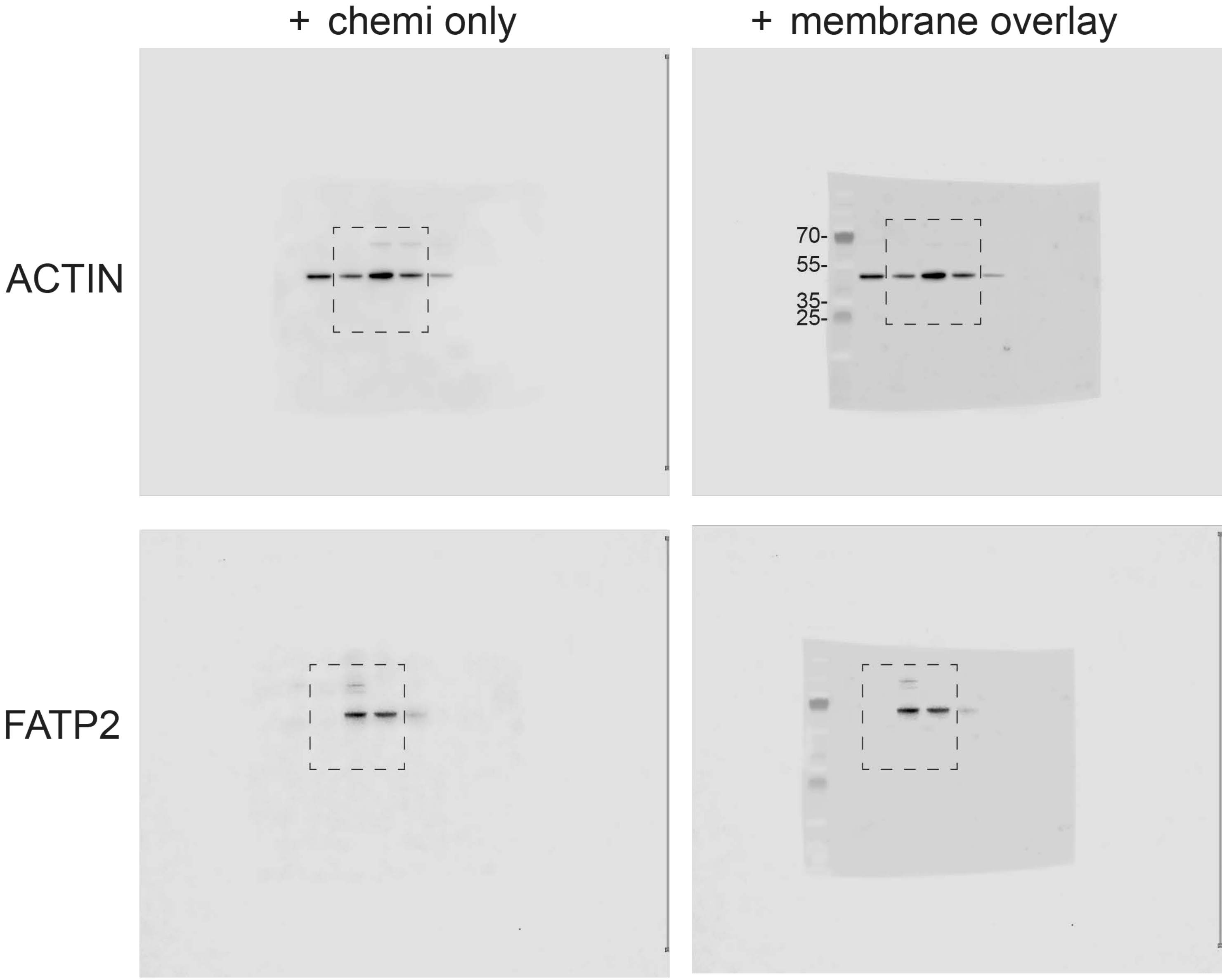

Final Figure

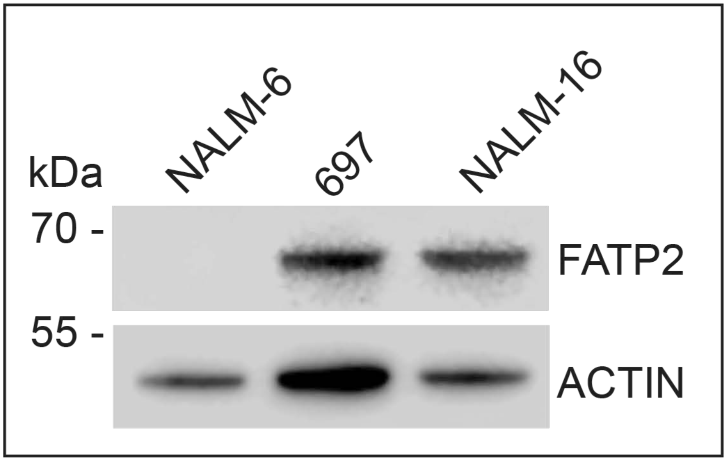

Supplementary Figure S4A Raw Blots

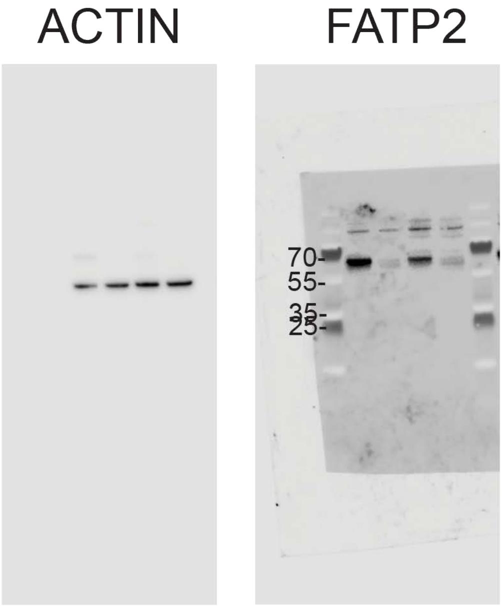

Final Figure

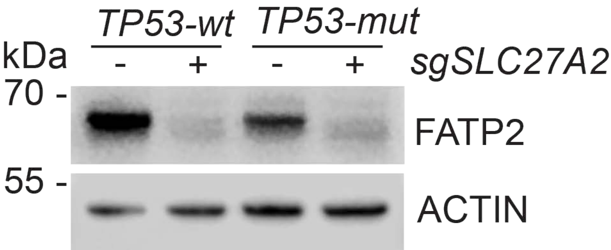

Supplement: Supplementary file 2 — Raw Western Blots [file 41375_2026_3030_MOESM2_ESM.pdf]
